# Seven new species within western Atlantic *Starksia atlantica*, *S. lepicoelia*, and *S. sluiteri* (Teleostei, Labrisomidae), with comments on congruence of DNA barcodes and species

**DOI:** 10.3897/zookeys.79.1045

**Published:** 2011-02-03

**Authors:** Carole C. Baldwin, Cristina I. Castillo, Lee A. Weigt, Victor Benjamin C.

**Affiliations:** 1 National Museum of Natural History, Smithsonian Institution, Washington, D.C. 20560; 2 Ocean Science Foundation, 4051 Glenwood, Irvine, CA 92604 and Guy Harvey Research Institute, Nova Southeastern University, 8000 North Ocean Drive, Dania Beach, FL 33004

**Keywords:** *Starksia*, DNA Barcoding, new species, species complex, biogeography

## Abstract

Specimens of Starksia were collected throughout the western Atlantic, and a 650-bp portion of the mitochondrial gene cytochrome oxidase-*c* subunit I (COl) was sequenced as part of a re-analysis of species diversity of western Central Atlantic shorefishes. A neighbor-joining tree constructed from the sequence data suggests the existence of several cryptic species. Voucher specimens from each genetically distinct lineage and color photographs of vouchers taken prior to dissection and preservation were examined for diagnostic morphological characters. The results suggest that Starksia atlantica, Starksia lepicoelia, and Starksia sluiteri are species complexes, and each comprises three or more species. Seven new species are described. DNA data usually support morphological features, but some incongruence between genetic and morphological data exists. Genetic lineages are only recognized as species if supported by morphology. Genetic lineages within western Atlantic Starksia generally correspond to geography, such that members of each species complex have a very restricted geographical distribution. Increasing geographical coverage of sampling locations will almost certainly increase the number of Starksia species and species complexes recognized in the western Atlantic. Combining molecular and morphological investigations is bringing clarity to the taxonomy of many genera of morphologically similar fishes and increasing the number of currently recognized species. Future phylogenetic studies should help resolve species relationships and shed light on patterns of speciation in western Atlantic Starksia.

## Introduction

The description of six new species of Caribbean Starksia by Williams and Mounts (2003) capped more than 100 years of systematic research on this New World labrisomid genus. It would have been reasonable to assume after such effort that there is little about the group left to discover. But the utilization of modern DNA barcoding techniques in taxonomic studies is revealing the need to reanalyze existing species classifications of many groups of animals and, in combination with traditional morphological analyses, resulting in the recognition of numerous new species (e.g., Crawford et al. 2010, Hebert et al. 2004, Pauls et al. 2010, Pöppe et al. 2010, Ward et al. 2008, Zemlak et al. 2009). Western Atlantic shorefishes are no exception (e.g., Tornabene et al. 2010; Victor 2007, 2010). Particularly for small cryptic reef fishes such as Starksia blennies, we do not know where we stand in terms of understanding species diversity, and our current concepts may be surprisingly incomplete.

Starksia fishes inhabit shallow to moderately deep (to ca. 30 m) rock and coral reefs in the western Central Atlantic and eastern Pacific oceans. They are small (Atlantic species are generally < 40 mm SL) and cryptic, but they often exhibit bright orange or red coloration in life. Twenty-one species are currently recognized in the western Atlantic (Williams and Mounts 2003), six of which are considered members of the Starksia ocellata species complex (Greenfield 1979).

The purpose of this paper is to describe the systematic results of our recent genetic and morphological investigations of western Atlantic Starksia, work that was prompted by our discovery of incongruences between preliminary genetic data and the current species classification. We describe seven new species within Starksia atlantica, Starksia lepicoelia, and Starksia sluiteri and provide keys to the species of each of those species complexes. We provide photographs of living and preserved pigment patterns to help in future identifications of the included species and in distinguishing them from western Atlantic Starksia species likely to be discovered in the future. Finally, we discuss geographical distributions of Starksia species and comment on congruence between DNA barcoding data and morphologically recognizable species.

## Materials and methods

Specimens used in this study were collected from Barbados, Belize, Bahamas, Curacao (Netherland Antilles), Florida, Honduras, Panama (Atlantic), Saba Bank (Netherland Antilles), St. Thomas (U.S. Virgin Islands), Tobago (Trinidad and Tobago), and Turks and Caicos. That material and additional museum specimens examined are listed in the appropriate species and comparisons sections. Starksia specimens included in the genetic analysis but not in the species accounts are tabulated in Appendix 1. Institutional abbreviations for collections follow Sabaj Pérez (2010).

Specimens were collected with quinaldine sulfate, rotenone, or clove oil using snorkel gear or scuba depending on depth. Field protocol involved taking digital color photographs of fresh color patterns and subsequently a tissue sample (muscle, eye, or fin clip) for genetic analysis. For many, particularly small specimens, it was necessary to remove the posterior 1/3 to 1/2 of the body to obtain enough tissue for genetic analysis. Voucher specimens were preserved and later used to investigate diagnostic morphological features of each recovered genetic lineage. Field measurements of standard length (SL), to the nearest 0.5 mm, were made by viewing specimens against a plastic ruler under a dissecting microscope. Lengths of voucher specimens generally were not re-measured in the lab because many vouchers are now incomplete specimens. Those that were measured in the lab were measured to the nearest 0.1 mm with digital calipers or with the aid of an ocular micrometer in a dissecting microscope. Lengths of head (HL) and genital papilla were measured to the nearest 0.1 mm with the same ocular micrometer and microscope. To ensure that we were not introducing bias due to shrinkage of specimens after preservation, head length as a percentage of SL was calculated only for specimens in which both measurements were made from preserved specimens. Counts of dorsal-, anal-, and caudal-fin rays were made from digital radiographs of specimens, from preserved specimens, or from photographs of voucher specimens taken prior to dissection. We followed Böhlke and Springer (1961) in counting the last two segmented rays of the dorsal and anal fins separately. Lateral-line scales were not counted because too many scales are missing on most specimens. This is likely due to the long time the specimens were held for processing prior to preservation and the physical manipulation of the specimens during processing. Pores from the circumorbital ossifications are either uniserial or paired; the positions of any paired pores are described based on their position relative to the orbit as though it were a clock; on the left side, for example, a pair of pores at 3 o’clock is on the posterior margin of the orbit, a pair at 6 o’clock is on the ventral margin.

Molecular techniques employed at the Smithsonian are as described below. Methods utilized to sequence DNA from specimens from Barbados, Honduras, Panama, and St. Thomas are as outlined in Victor (2010). Tissue samples for molecular work were stored in saturated salt buffer (Seutin et al. 1990) or in 95% ethanol. Genomic DNA was extracted from up to approximately 20 mg minced preserved tissue via an automated phenol:chloroform extraction on the Autogenprep965 (Autogen, Holliston, Massachusetts) using the mouse tail tissue protocol with a final elution volume of 50 µL. For polymerase chain reaction (PCR), 1 µL of this genomic DNA was used in a 10 µL reaction with 0.5 U Bioline (BioLine USA, Boston, Massachusetts) Taq polymerase, 0.4 µL 50 mM MgCl2, 1 µL 10× buffer, 0.5 µL 10 mM deoxyribonucleotide triphosphate (dNTP), and 0.3 µL 10 µM each primer FISH-BCL (5’-TCAACYAATCAYAAAGATATYGGCAC) and FISH-BCH (5’-TAAACTTCAGGGTGACCAAAAAATCA). The thermal cycler program for PCR was 1 cycle of 5 min at 95°C; 35 cycles of 30 s at 95°C, 30 s at 52°C, and 45 s at 72°C; 1 cycle of 5 min at 72°C; and a hold at 10°C. The PCR products were purified with Exosap-IT (USB, Cleveland, OH) using 2 µL 0.2× enzyme and incubated for 30 min at 37°C. The reaction was then inactivated for 20 min at 80°C. Sequencing reactions were performed using 1 µL of this purified PCR product in a 10 µL reaction containing 0.5 µL primer, 1.75 µL BigDye buffer, and 0.5 µL BigDye (ABI, Foster City, California) and run in the thermal cycler for 30 cycles of 30 s at 95°C, 30 s at 50°C, 4 min at 60°C, and then held at 10°C. These sequencing reactions were purified using Millipore Sephadex plates (MAHVN-4550; Millipore, Billerica, Massachusetts) per manufacturer’s instructions and stored dry until analyzed. Sequencing reactions were analyzed on an ABI 3730XL automated DNA sequencer, and sequence trace files were exported into Sequencher 4.7 (GeneCodes, Ann Arbor, MI). Using the Sequencher program, ends were trimmed from the raw sequences until the first and last 10 bases contained fewer than 5 base calls with a confidence score (phred score) lower than 30. After trimming, forward and reverse sequences for each specimen were assembled. Each assembled pair was examined and edited by hand, and each sequence was checked for stop codons. Finally the consensus sequence (655 bp) from each contig was aligned and exported in a nexus format (sensu Swofford 2002).

A neighbor-joining tree (Saitou and Nei 1987) and distance matrix were generated using Paup*4.1 (Swofford 2002) on an analysis of Kimura two-parameter distances (Kimura 1980). The neighbor-joining tree is not intended to reflect phylogenetic relationships. The labels for each entry on the tree is our DNA number, and we include that number in the material examined sections and figure captions. Abbreviations used in DNA numbers reflect geographical location: BAH – Bahamas, BAR – Barbados, BLZ – Belize, BRZ – Brazil, CUR – Curacao, FLA – Florida, HON – Honduras, PAN – Panama, SAB – Saba Bank (Netherland Antilles), STVI – St. Thomas Virgin Islands, TCI – Turks and Caicos, TOB – Tobago. COI sequences are deposited in Genbank (accession numbers HQ543038-HQ543055, HQ571151-HQ571164, HQ600864-HQ600963).

## Results

**Figure 1.**
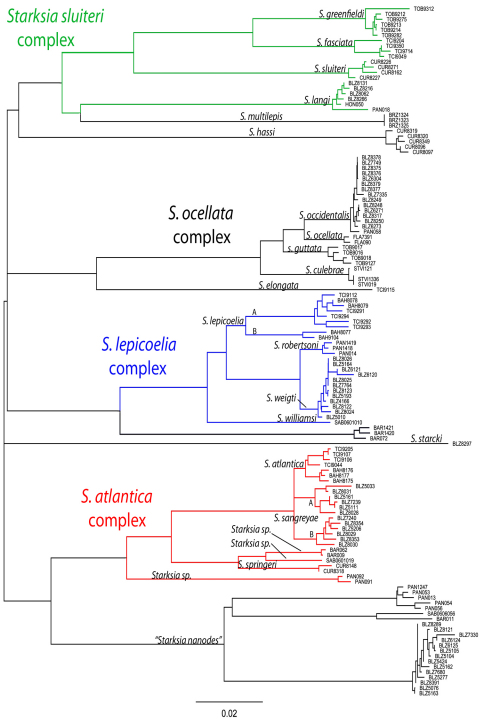
Neighbor-joining tree derived from cytochrome *c* oxidase I sequences showing genetically distinct lineages of western Atlantic Starksia.

A neighbor-joining tree derived from western Atlantic Starksia COl sequences is shown in Fig. 1. Thirteen of the 21 currently recognized western Atlantic Starksia species are represented in the tree: Starksia atlantica, Starksia culebrae, Starksia elongata, Starksia fasciata, Starksia guttata, Starksia hassi, Starksia lepicoelia, Starksia multilepis, Starksia nanodes, Starksia occidentalis, Starksia ocellata, Starksia sluiteri, and Starksia starcki. Four species, Starksia culebrae from the U.S. Virgin Islands*, S. guttata* from Tobago, Starksia occidentalis from Belize, and Starksia ocellata from Florida, cluster on the tree but represent genetically distinct lineages. Those results support Greenfield’s (1979) recognition of a Starksia ocellata species complex with several allopatric component species. Similarly, Starksia atlantica, Starksia lepicoelia, Starksia nanodes, and Starksia sluiteri comprise multiple, geographically distinct, genetic lineages, suggesting that they also represent species complexes comprising multiple allopatric species. We do not deal further with the Starksia nanodes complex in this paper because no genetic data is available from the type locality, Bahamas, and we are thus uncertain if any of the four genetic lineages on the tree (Barbados, Belize, Panama, and Saba Bank) represents Starksia nanodes Böhlke & Springer 1961. We also do not deal further with five species, Starksia elongata, Starksia fasciata, Starksia hassi, Starksia multilepis, and Starksia starcki (but see discussion of Starksia fasciata under the Starksia sluiteri complex section). Each of those speciesis represented in our material from only one geographical location, and material from additional geographic locations is needed to determine if they represent species complexes. We note that our material of Starksia elongata, Starksia fasciata, Starksia hassi, and Starksia multilepis is from the type localities of those species or relatively close by; the type locality of Starksia starcki, however, is Florida, and our specimen is from Belize.

The multiple genetic lineages within Starksia atlantica, Starksia lepicoelia, and Starksia sluiteri are the focus of the species treatments below. For each complex, we discuss congruence of the component genetic lineages with results of our morphological investigation. When diagnostic morphological features (primarily pigment) support the genetic data, we recognize genetic lineages as species. Greenfield (1979) noted that the ability to identify individuals of the Starksia ocellata complex to species based on morphology without prior knowledge of locality supports the recognition of the component populations as species vs. subspecies. We concur, and believe that the addition of the COl data strengthens this argument. There are no available names for new species within any of Starksia atlantica, Starksia lepicoelia, and Starksia sluiteri complexes, and the seven unnamed species discovered are described herein as new. Keys to the species of the Starksia atlantica, Starksia lepicoelia, and Starksia sluiteri complexes are provided. We suggest that readers use the taxonomic key to western Atlantic Starksia provided by Williams and Mounts (2003) to identify Starksia atlantica, Starksia lepicoelia, and Starksia sluiteri and the keys in this paper to distinguish the members within each complex. Note that the sixth couplet of the Williams and Mounts (2003) key contains an error: 6b should lead the user to couplet 10, not 9 as indicated. The geographical locations listed for each species in our keys are the type locality plus any additional localities for which we have genetic data. Additional collecting and study are needed to determine the distributions of all western Atlantic Starksia species. Distance matrices for intra- and interspecific variation in COl sequences for the Starksia atlantica, Starksia lepicoelia, and Starksia sluiteri species complexes are provided in tables within the text. A distance matrix for all lineages is in Appendix 2.

## Starksia atlantica Species Complex

Longley (1934) described Starksia atlantica from a single specimen from Andros Island, Bahamas. The neighbor-joining tree derived from COI sequences (Fig. 1) includes five distinct genetic lineages in the Starksia atlantica complex. The lineages from Barbados (BAR) and Panama (PAN) are known only from larvae or juveniles and are not discussed further. The Panama lineage is highly divergent in COl, and it likely represents a cryptic species within Starksia atlantica or one of the eight western Atlantic Starksia species not identified in our material. The other three lineages—Curacao (CUR), Saba Bank (SAB), and Bahamas/Turks and Caicos/Belize (BAH/TCI/BLZ) comprise specimens originally identified as Starksia atlantica on the basis of absence of an orbital cirrus. (Note: Williams and Mounts (2003) correctly noted the absence of an orbital cirrus as diagnostic for Starksia atlantica in their key to western Atlantic Starksia [p. 147], but they erroneously stated “orbital cirri present” intheir treatment of the species [p. 160].) Within the BAH/TCI/BLZ lineage, there are three sublineages, two from Belize and one from Bahamas/Turks & Caicos Islands (or four if the latter is viewed as two). We have identified the specimens from Bahamas and Turks and Caicos as Starksia atlantica (Longley) based on the type locality (Bahamas) and pigment pattern, specifically the presence of two or three rows of block-like blotches on the trunk that are irregular in size and shape (Böhlke and Springer 1961). We found no consistent differences between specimens from the Bahamas and Turks and Caicos.

The two Belize sublineages differ from other members of the Starksia atlantica complex by the presence of regular, vertical, brown bars on the trunk separated by narrow white interspaces and a well-defined horseshoe-shaped blotch on the cheek. Although those two sublineages are genetically similar to Starksia atlantica, we recognize the two lineages from Belize as a distinct species based on their strikingly different pigment pattern and geographic separation. We found no consistent morphological variation between the two Belize sublineages and treat them as a single new species. Two specimens of this new species were illustrated as Starksia atlantica by Greenfield and Johnson (1981: Fig. 3A,B), who noted consistent differences in pigmentation on the body between their material from Belize and Honduras and the description of pigmentation for Starksia atlantica by Böhlke and Springer (1961). The other two genetic lineages of Starksia atlantica (Fig. 1) are from Curacao (CUR) and Saba Bank (SAB). The Curacao specimens have a distinctive pattern of pigment on the cheek and pectoral-fin base, and we recognize them as a distinct species. The single sequence from Saba Bank likely represents a new species (Fig. 1), but additional material is needed to confidently assess its status (see “Remarks” under “*Starksia sp.*” below). We describe two new species within the Starksia atlantica complex, Starksia sangreyae from Belize and Starksia springeri from Curacao.

### Starksia sangreyae

Castillo & Baldwin sp. n.

urn:lsid:zoobank.org:act:F61A042F-F042-48EA-B4E1-C7AD79866916

Figs 1 2 4 Table 1

Starksia atlantica , Greenfield and Johnson (1981), Fieldiana Zoology 8: Fig. 3A–B (black and white drawings of male and female specimens from Belize)

#### Type Locality:

Belize, Central America

#### Holotype.

USNM 398932, BLZ 5111, male, 16.0 mm SL, sta. CB05-9, south side of island, Carrie Bow Cay, Belize, 1–2 m, 25 April 2005, C. Baldwin, D. Smith, L. Weigt, J. Mounts (small fillet removed from right side for DNA tissue sampling).

#### Paratypes (all Belize).

Note – posterior portion of body destroyed for DNA tissue sampling of all paratypes except USNM 276147 and 321073, which are not DNA vouchers. USNM 398939, BLZ 8031, female, 18.0 mm SL, sta. CB08-2, sand bottom and coral heads, Curlew Cay, 16°47'24.1"N, 88°04'41.0"W, 5–8 m, 15 May 2008. USNM 398933, BLZ 5033, female, 16.5 mm SL, sta. CB05-3, spur and grove, Carrie Bow Cay, 9–22 m, 22 April 2005. USNM 398936, BLZ 8028, male, 17 mm SL, sta. CB08-2 (see CB08-2 above). USNM 398934, BLZ 5161, female, 17.0 mm SL, sta. CB05-12, Curlew Cay, 15–21 m, 27 April 2005. USNM 398935, BLZ 5206, female, 12.0 mm SL, sta. CB05-13, Belize (no other collection data available), 29 April 2005. USNM 398937, BLZ 8029, male, 17.0 mm SL, sta. CB08-2 (see CB08-2 above). USNM 398938, BLZ 8030, female, 19.0 mm SL, sta. CB08-2 (see CB08-2 above). USNM 398940, BLZ 8353, female, 16.0 mm SL, sta. CB08-32, Tobacco Cay, 16°53'23.8"N, 88°03'53.8"W, 0–5 m, 25 May 2008. USNM 276147, male, 15.0 mm SL, sta. GDJ 84-14, off northwest end of Carrie Bow Cay, 2–3 m, 7 Nov 1984. USNM 321073, female, 18.0 mm SL, sta. GDJ 90-2, reef flat and crest, coral rubble and sand substrate, Carrie Bow Cay, 3–6 ft., 18 Sep 1990.

#### Additional Material (not DNA vouchers).

Belize: USNM 398943, 4 specimens; USNM 398944, 2; USNM 398945, 4; USNM 321066, 1; USNM 276068, 1; USNM 398941, 1; USNM 398942, 1.

#### Diagnosis.

A species of Starksia distinguished by the following combination of characters: no orbital cirrus, regular vertical brown bars on trunk separated by narrow white interspaces, and a well defined horseshoe-shaped blotch of dark pigment on cheek.

#### Description.

See Table 1. Dorsal spines XIX–XX, usually XIX (XIX in holotype); segmented dorsal rays 7–8 (8); total dorsal elements 26–27, usually 27 (27); anal spines II; segmented anal rays 14–16, usually 15 (15); dorsal segmented caudal-fin rays 7; ventral segmented caudal-fin rays 6; dorsal procurrent caudal-fin rays 5–6, usually 6 (6); ventral procurrent caudal-fin rays 4–6, usually 5 (5); segmented pelvic-fin rays 2; pectoral-fin rays 14–15, rarely 15 (14); vertebrae 10+21–22= 31–32, rarely 31(10+22=32); 1–4 pairs of infraorbital pores, usually 4 pairs between 3 and 6 o’clock (4 pairs); orbital cirri absent; nape cirri present; anterior nostril cirri present; belly and pectoral-fin base naked or with only a few rows of scales anterior to the anus.

**Figure 2.**
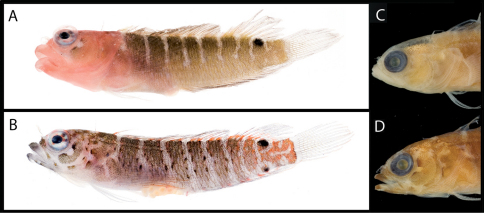
Male and female color patterns of Starksia sangreyae: **A** USNM 398932, holotype, BLZ 5111, 16.0 mm SL, male **B** USNM 398933, BLZ 5033, 16.5 mm SL, female. **C–D** Diagnostic patterns of cheek pigment of preserved female and male – **C** USNM 276147, 15.0 mm SL, male **D** USNM 321073, 18.0 mm SL, female. Photographs by Carole Baldwin, Cristina Castillo, Donald Griswold, and Julie Mounts.

**Table 1.** Frequency distributions of counts among species of the Starksia atlantica complex.

	*Dorsal Spines*			*Dorsal Soft Rays*		*Total Dorsal*		*Anal Soft Rays*			
	XVIII	XIX	XX	7	8	26	27	14	15	16	
Starksia springeri	1*	-	-	-	1*	1*	-	-	1*	-	
Starksia sangreyae	-	11*	2	7	6*	5	8*	1	9*	2	
Starksia atlantica 1	1	7*	-	2	6*	3	5*	-	4	4*	
	*Pectoral Rays*			*Dorsal Procurrent Caudal Rays*		*Ventral Procurrent Caudal Rays*			*Vertebrae*		
	13	14	15	5	6	4	5	6	31	32	33
Starksia springeri	*-*	3*	-	1*	-	-	1*	-	-	1*	-
Starksia sangreyae	-	20*	1	3	7*	1	8*	1	1	12*	-
Starksia atlantica 1	1	8	1	4	4	-	4	4	2	5	1

* Indicates count of holotype1 Longley (1934) did not provide counts of pectoral-fin rays or vertebrae for the holotype of Starksia atlantica

Specimens examined ranging from 12.0 to 19.0 mm SL; HL 29–34% SL (31% in holotype); male genital papilla adhered to first anal spine proximally; papilla length between two-thirds and three-quarters length of first anal spine, 0.6–1.0 mm; some females with very small genital papilla.

#### Pigment.

Vertical brown bars present on trunk separated by narrow white interspaces; anteriormost 6 bars relatively uniform in all specimens; posterior bars often irregular or incompletely formed. A thick horseshoe-shaped blotch of pigment present on cheek. Bright orange pigment present on distal portions of pectoral-fin rays, and pale orange pigment usually present on distal portions of posterior anal-, caudal-, and soft dorsal-fin rays. Color pattern sexually dimorphic: males with pale red heads (vs. females without red coloration); relatively poorly defined horseshoe-shaped blotch of pigment on cheek that fades posteriorly (well-defined horseshoe-shaped blotch on cheek that is sometimes mirrored on operculum and pectoral fin base); body bars tan and usually with some gold or green color in life (darker and without green/gold color but some posterior bars often with some orange pigment); body bars usually terminating ventrally dorsal to ventral midline (body bars usually extending to ventral midline); blotches of tan/gold pigment on base of dorsal fin associated with body bars, and no tan/gold color present on anal fin (bright orange markings on base of dorsal fin associated with body bars and several bright orange spots on base of anal fin); and large dark spot, roughly diameter of pupil or larger, on trunk at posterior end of dorsal fin (two large dark spots on trunk, one at posterior end of dorsal fin similar in size to that of males, and smaller spot at posterior end of anal fin).

#### Color in preservative.

Vertical bars on trunk, horseshoe-shaped blotch of pigment on cheek, and spot at posterior end of dorsal fin (and anal fin in females) retained in preservative; margins of at least some body bars in females with small dark spots; prominent patches of melanophores on jaws and gular region, and scattered pigment (heavier in females) on rest of head; dorsal fin ranging from overall dusky to having concentrations of pigment on base of fin associated with body bars; caudal fin with light pigment on outer rays, and pectoral fin with scattered melanophores over entire fin; pelvic fin clear.

#### Etymology.

The species name is in honor of Mary Sangrey for her many years of work coordinating the intern program at the Smithsonian’s National Museum of Natural History. Mary brought the intern application of the second author to the first author’s attention and took the first steps toward procuring funding for Castillo’s internship.

#### Distribution.

All material that we examined is from Belize. The range of the species also apparently includes Honduras, as Greenfield and Johnson (1981) noted that a specimen of Starksia atlantica from Honduras has regular vertical bars of pigment on the body.

### Starksia springeri

Castillo & Baldwin sp. n.

urn:lsid:zoobank.org:act:495CE72B-82CD-4A2B-B192-ACAA389F40FC

Figs 1 3 4 Table 1 

#### Type Locality:

Curacao, Netherland Antilles

#### Holotype.

USNM 398945, female, 19.0 mm SL, sta. CUR08-10, Blue Bay, Curacao, 12°07'59.22"N, 68°59'05.34"W, 1–25 m, 17 March 2008, C. Baldwin, D. Smith, L. Weigt (not a DNA voucher).

#### Paratypes (all Curacao).

USNM 399658, CUR 8148, male(?), 15.0mm SL, sta. CUR08-03, Cas Abou, 12°13'34.04"N, 69°05'29.95"W, 0–4 m, 12 March 2008, (posterior portion of body destroyed for DNA tissue sampling). USNM 399659, CUR 8318, (sex unknown), 12.0 mm SL, sta. CUR08-05, Blue Bay, 12°07'57.14"N, 68°59'06.03"W, 0–25 m, 14 March 2008, (posterior portion of body destroyed for DNA tissue sampling).

#### Diagnosis.

A species of Starksia distinguished by the following combination of characters: no orbital cirrus; trunk with irregular dark blotches on pale background; pectoral-fin base with relatively straight margins defining pale gap that separates two dark blotches; cheek with distinctive dark and pale markings: anterior portion of cheek with prominent dark blotch, anteroventral and posterior margins of blotch well defined by pale regions; posterior pale area on cheek bordered posteriorly by thin, dark, anteroventral-to-posterodorsal streak of pigment along distal edge of preopercle.

#### Description.

See Table 1**.** The female holotype is the only complete specimen available. Counts in parentheses are those for the holotype. Few counts could be made on partial specimens; when available, counts of partial specimens precede those of holotype. Dorsal spines (XVIII); segmented dorsal rays (8); total dorsal elements (26); anal spines (II); segmented anal rays (15); dorsal segmented caudal-fin rays (7); ventral segmented caudal-fin rays (6); dorsal procurrent caudal-fin rays (5); ventral procurrent caudal-fin rays (5); segmented pelvic-fin rays 2 (2); pectoral-fin rays 14 (14); vertebrae (10+22= 32); infraorbital pores paired or unpaired, usually 1–3 pairs (3 pairs); if only one pair of pores, pair situated at 3 o’clock; 3 pairs in holotype located at 3, 5, and 6 o’clock; orbital cirri absent; nape cirri present; anterior nostril cirri present; belly and pectoral-fin base completely naked.

**Figure 3.**
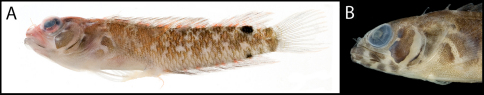
**A** Color pattern of Starksia springeri, USNM 399658, CUR 8148, paratype, 15.0 mm SL, male(?) **B** diagnostic pigment pattern on cheek and pectoral-fin base in preserved Starksia springeri, USNM 398945, holotype, 19.0 mm SL, female. Photographs by Carole Baldwin, Cristina Castillo, and Donald Griswold.

**Figure 4.**
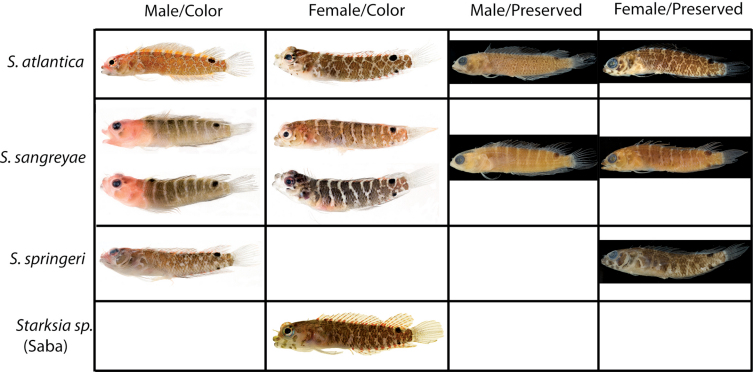
Comparisons among species of the Starksia atlantica complex. Left to right for each row -- Starksia atlantica: AMNH 241247; USNM 399621, BAH 8176, 15.0 mm SL; USNM 386971, 19.0 mm SL; USNM 386242, 17.0 mm SL. Starksia sangreyae: (Note: top and bottom images in first two columns represent Starksia sangreyae A and Starksia sangreyae B genetic sublineages, respectively.) Males – USNM 398936 (top), paratype, BLZ 8028, 17.0 mm SL and USNM 398937 (bottom), paratype, BLZ 8029, 17 mm SL; Females – USNM 398934 (top), paratype, BLZ 5161, 17.0 mm SL and USNM 398940 (bottom), paratype, BLZ 8353, 16.0 mm SL; preserved – USNM 276147, paratype, 15.5 mm SL; USNM 321073, paratype, 18.0 mm SL. Starksia springeri: USNM 399658, paratype, CUR 8148, 15.0 mm SL; USNM 398945, holotype, CUR 08-10, 19.0 mm SL; Starksia sp. (Saba): SABA-06-01, 15.0 mm SL (no voucher).Photographs by Carole Baldwin, Cristina Castillo, Donald Griswold, Julie Mounts, Ross Robertson, James Van Tassell, and Jeffrey Williams.

Specimens examined ranging from 12.0 to 19.0mm SL; HL 25–32% SL (32% in holotype); genital-papilla length in 15.0-mm SL paratype 0.3 mm, one-fourth length of first anal spine (broken); papilla adhered to spine proximally. Note: the presence of a small but measurable genital papilla on 15.0-mm SL paratype suggests that it is a male: although female Starksia sometimes have a small genital papilla, the 19 mm female holotype does not. As noted below, the 15 mm paratype has a pupil-size dark spot at posterior base of anal fin, which usually characterizes females. We tentatively recognize this paratype as a male.

#### Pigment.

(Note: a field photograph of the 12.0-mm SL paratype is a dorsal view of poor quality, and only the head remains as a preserved voucher. The following description is based on the 15.0-mm SL paratype and the 19.0-mm SL holotype.) Trunk with irregular dark blotches on pale background, most blotches consisting of orange chromatophores and melanophores in paratype; two dark spots present on trunk, large one at posterior end of dorsal fin (larger than pupil diameter) and smaller spot at posterior end of anal fin. Paratype with pale orange and brown pigment on head; tips of jaws with dark pigment in both paratype and holotype, but rest of jaws and gular region distinctly barred in holotype, mottled with tiny spots in paratype; cheek with distinctive dark and pale markings: anterior portion of cheek with prominent dark blotch, its anteroventral and posterior margins well defined by pale regions; posterior pale area on cheek bordered posteriorly by thin, dark, anteroventral-to-posterodorsal streak of pigment along distal edge of preopercle. Bright orange markings present on bases of dorsal fin and anal fins, sometimes occurring in pairs; bright orange pigment also present on distal portions of pectoral-fin rays; pale orange pigment present distally on at least some rays of soft dorsal, caudal, and anal fins; pectoral-fin base with relatively pale gap separating two dark blotches, margins of gap relatively straight; dark blotches on pectoral-fin base comprising orange chromatophores and melanophores.

#### Color in preservative.

(Note: pigmentation on trunk in preservative based on the only entire specimen, female holotype.) Trunk with irregular dark blotches on pale background; spots at posterior ends of dorsal and anal fins retained in preservative. Dark markings on head described above retained in preservative, mottled jaws and gular region of male(?) paratype strikingly different from barred markings on female holotype; top of head in both specimens covered with scattered melanophores; dark and pale regions on cheek and pectoral-fin base retained in preservative. Anal and pectoral fins with lightly scattered melanophores; caudal fin with light pigment on outer rays; pelvic fin clear.

#### Etymology.

Named in honor of Victor G. Springer, Senior Scientist Emeritus, Smithsonian National Museum of Natural History, for his contributions to the systematics of blennioid fishes, including Starksia, and for advice and friendship he has bestowed upon the first author.

#### Distribution.

Known only from Curacao, Netherland Antilles.

### Starksia sp.

Figs 1 4 

#### Locality:

**Saba Bank, Netherland Antilles**

#### Material Examined.

Specimen and photograph: USNM 388032, sta. SABA-06–25, 9.0 mm female (not a DNA voucher), near Coral Garden at SE edge of Saba Bank, Netherland Antilles, 17°21.10'N, 63°15.08'W, 15–18 m, 4 Jan 2006; photograph: 15.1 mm SL female (not a DNA voucher), sta. SABA-06-01, Saba Bank just south of Poison Bank, Netherland Antilles, 17°28.47'N, 63°13.40'W, 24–27 m, 4 Jan 2006 (photographs by Jeffrey T. Williams).

#### Remarks.

A DNA sequence from a single specimen collected at Saba Bank (Netherland Antilles) is genetically distinct from the other members of the Starksia atlantica species complex (SAB 0601019, Fig. 1). Our material includes color photographs of 9.0- and 15.1-mm SL females and the preserved 9.0 mm specimen (USNM 388032). Presumably the 9.0 and 15.1 mm specimens are the same species as the specimen represented on the tree, but we do not have tissue samples of either for genetic analysis or a preserved voucher of SAB 0601019 for morphological analysis.

Trunk pigment in the images and preserved specimen is similar to that of Starksia atlantica from the Bahamas and Starksia springeri from Curacao (i.e., mottled vs. barred as in Starksia sangreyae), but the Saba specimens lack the horseshoe-shaped blotch of pigment on the cheek characteristic of Starksia atlantica and the distinctive dark and pale markings on the cheek of Starksia springeri. The blotches of trunk pigment in the Saba Bank specimens are neither conspicuously block-like nor clearly organized in horizontal tiers as they are in Starksia atlantica. Specimens from Saba Bank presumably represent another new species within Starksia atlantica, but additional specimens are needed for comparative purposes and description.

### Comparisons among Species of the Starksia atlantica Complex (Figs 4–5, Table 1)

**Comparative material.** Starksia atlantica. Bahamas: USNM 386971, 1 specimen (not a DNA voucher); USNM 386580, 1 (not a DNA voucher); USNM 386242, 6 (not DNA vouchers); USNM 399619, 3 (not DNA vouchers); USNM 399620, BAH 8175; USNM 399621, BAH 8176; USNM 399622, BAH 8177. Turks and Caicos Islands: USNM 399643, TCI 9044; USNM 399644, TCI 9106; USNM 399645, TCI 9107; USNM 399647, TCI 9205. Navassa Island: USNM 360422, 3; USNM 360194, 2; USNM 359543, 2; USNM 360210, 3.

Members of the Starksia atlantica complex are diagnosed by the absence of an orbital cirrus. Starksia sangreyae is distinct in having regular vertical body bars separated by narrow pale interspaces and a well-defined horseshoe-shaped blotch on the cheek. Starksia springeri, Starksia atlantica, and the specimens from Saba Bankhave irregular dark blotches on a pale background on the trunk, the blotches better defined in our Starksia atlantica material than in the other species and often more clearly arranged in two or three horizontal tiers. Starksia springeri, Starksia atlantica, and the Saba Bank specimens can be distinguished on the basis of pigment patterns on the cheek: specimens from Saba Bank lack cheek blotches; Starksia atlantica has a horseshoe-shaped blotch on the cheek; and Starksia springeri has a prominent dark blotch on the cheek bordered anteroventrally and posteriorly by pale areas and a thin, dark, anteroventral-to-posterodorsal streak of pigment along the distal edge of the preopercle. Although Starksia sangreyae and Starksia atlantica are easily separated based on trunk pigment, we note that both have a horseshoe-shaped blotch of pigment on the cheek; the blotch is most prominent and best defined in Starksia sangreyae females, often completely faded in preserved Starksia sangreyae males. Starksia atlantica and Starksia springeri can be separated based on pigment on the pectoral-fin base: in Starksia atlantica, the pale gap between two blotches of darker pigment has wavy margins, whereas in Starksia springeri, the margins of the pale gap are relatively straight. Starksia springeri has XVIII dorsal spines vs. usually XIX in the other species (Table 1), but we have only one entire specimen of Starksia springeri on which to base counts. No other significant differences were found in numbers of fin rays or vertebrae among species of the Starksia atlantica complex.

A photograph of a specimen identified as Starksia atlantica from St. Croix, U. S. Virgin Islands (taken by William Smith-Vaniz) shows irregular block-like blotches on the body arranged in roughly 3 horizontal tiers, wavy margins on the pale gap that separates two darker areas on the pectoral-fin base, and an irregular horseshoe-shaped blotch of pigment on the cheek. The U.S. Virgin Islands are thus likely part of the geographical distribution of Starksia atlantica Longley. Several USNM specimens identified as Starksia atlantica from Navassa Island exhibit pigmentation that is somewhat intermediate between that of Starksia atlantica and Starksia sangreyae: bars of pigment are present on the trunk anteriorly as in Starksia sangreyae, but trunk pigment is more block-like posteriorly as in Starksia atlantica; Navassa specimens also have an irregular horseshoe-shaped blotch on the cheek as in Starksia atlantica. Further genetic and morphological investigation should help clarify species issues of Starksia atlantica from Navassa Island.

**Figure 5.**
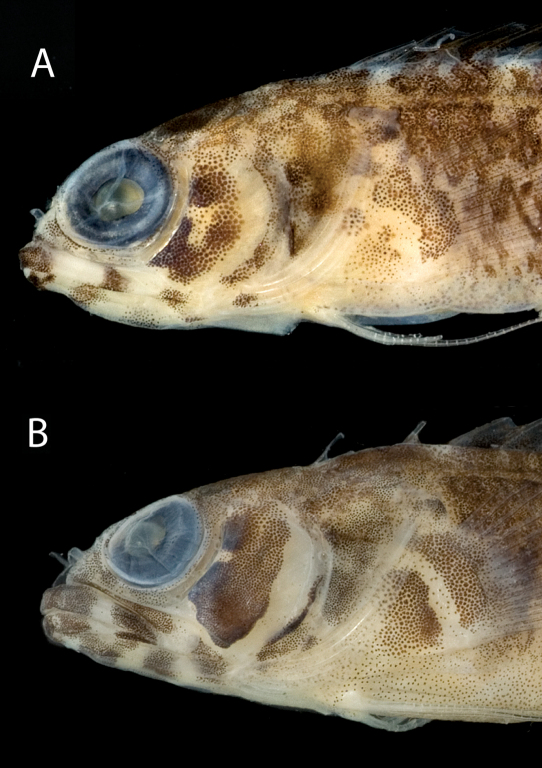
Diagnostic features in preserved **A** Starksia atlantica, USNM 386242, 17.0 mm SL, male—note irregular horseshoe-shaped blotch of pigment on cheek and wavy margins of pale gap on pectoral-fin base; and **B** Starksia springeri, USNM 398945, holotype, 19.0 mm SL, female—note pale regions at anteroventral and posterior margins of dark cheek blotch, thin dark anteroventral-to-posterodorsal streak of pigment along distal edge of preopercle, and relatively straight margins of pale gap on pectoral-fin base. Photographs by Cristina Castillo and Donald Griswold.

### Key to Species of the Starksia atlantica Complex

**Table:** 

1a	Body with vertical brown bars separated by narrow white interspaces	Starksia sangreyae (Belize)
1b	Body with irregular dark blotches on pale background	2
2a	Dark blotches on trunk often arranged in two or three horizontal tiers; pale gap between two blotches of darker pigment on pectoral-fin base with wavy margins; cheek with irregular horseshoe-shaped blotch of pigment; no streak of dark pigment along distal edge of preopercle	Starksia atlantica (Bahamas, Turks and Caicos)
2b	Dark blotches on trunk not conspicuously arranged in horizontal tiers; pale gap between two blotches of darker pigment on pectoral-fin base with straight margins; cheek with prominent dark blotch bordered anteroventrally and posteriorly by pale areas and a thin, dark, anteroventral-to-posterodorsal streak of pigment along distal edge of preopercle	Starksia springeri (Curacao)

## Starksia lepicoelia Species Complex

**Figure 6.**
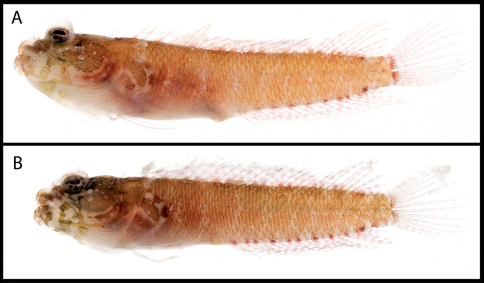
Comparison of Starksia lepicoelia specimens from Bahamas from genetically distinct lineages (see Fig. 1): **A** USNM 399615, BAH 8077, 25.0 mm SL, female **B** USNM 399617, BAH 8079, 19.0 mm SL, female. Photographs by Carole Baldwin.

Böhlke and Springer (1961) described Starksia lepicoelia on the basis of numerous specimens from the Bahamas and one from St. John, U.S. Virgin Islands. The presence of a simple cirrus above the eye, two externally obvious pelvic-fin rays, a completely scaled belly or at least posterior half scaled, and usually 17 anal-fin soft rays are diagnostic of the species. Six genetic lineages in our data set cluster in the Starksia lepicoelia complex (Fig. 1). There are no photographs or vouchers of the Barbados specimens (BAR on tree), and that lineage is not discussed further. Clearly it represents either a cryptic species within Starksia lepicoelia or one of the eight species of western Atlantic Starksia not identified in our material. Two of the Starksia lepicoelia lineages are from the Bahamas/Turks and Caicos (BAH/TCI), and although sequence divergence for the two is 4–6% -- much higher than typical intraspecific variation in western Atlantic Starksia – we were unable to find consistent morphological differences between them and tentatively recognize them together as Starksia lepicoelia (Fig. 6). A fourth genetic lineage comprises specimens from Belize (BLZ), and a fifth, specimens from Panama (PAN). Although those lineages differ by only about 1% sequence divergence in COl, they are easily distinguished by color pattern. We describe the specimens from Belize and Panama as two new species. A sixth genetic lineage is represented in our tree by a single specimen from Saba Bank, Netherland Antilles. Based on that specimen and several lots of non-voucher material, we recognize the Saba Bank population as a fourth species within the Starksia lepicoelia complex.

### Starksia weigti

Baldwin & Castillo sp. n.

urn:lsid:zoobank.org:act:91F47395-F5D4-4160-A645-5266D10E6DBB

Figs 1 7 10 11 Table 2 

#### Type Locality:

Belize, Central America

#### Holotype.

USNM 399648, BLZ 5010, male, 20.5 mm SL, sta. CB05-01, spur and groove, Carrie Bow Cay, Belize, 6–8 m, 21 Apr 2005, C. Baldwin, D. Smith, L. Weigt, J. Mounts (small fillet removed from right side for DNA tissue sampling).

#### Paratypes (all Belize).

USNM 399649, BLZ 5164, female, 19.0 mm SL, sta. CB05-12, Curlew Cay, 21–25 m, 27 Apr 2005, (posterior portion of body destroyed for DNA tissue sampling). USNM 399653, BLZ 8026, female, 17.5 mm SL, sta. CB08-02, sand bottom and coral heads, Curlew Cay, 16°47'24.1"N, 88°04'41.0"W, 5–8 m, 15 May 2008, (posterior portion of body destroyed for DNA tissue sampling). USNM 399652, BLZ 8025, female, 18.0 mm SL, sta. CB08-02, same collection information as above, (posterior portion of body destroyed for DNA tissue sampling). USNM 399651, BLZ 8024, female, 19.0 mm SL, sta. CB08-02, same collection information as above, (posterior portion of body destroyed for DNA tissue sampling). USNM 399654, CB08-2, 2 specimens: (1) 19.5 mm SL female, (1) 19.0 mm SL female (not DNA vouchers), same collection information as above. USNM 399656, BLZ 8123, juvenile, 9.5 mm SL, sta. CB08-10, east wall drop off, Glovers Cay, 16°42'36.1"N, 87°51'05.3"W, 11–23 m, 18 May 2008, (posterior portion of body destroyed for DNA tissue sampling). USNM 399655, BLZ 8122, female, 18.0 mm SL, sta. CB08-10, same collection information as above, (posterior portion of body destroyed for DNA tissue sampling). USNM 274922, Sta. K-103, 2 females, 20.0 and 24.0 mm SL, spur and groove, Carrie Bow Cay, 6–8 m, 10 June 1981. USNM 276063, Sta. GDJ 84-8, 2 males, 20.5 and 23.0 mm SL, Carrie Bow Cay, 24–30 m, 5 Nov 1984.

#### Additional Material (not DNA vouchers, all Belize).

USNM 399650, BLZ 5193, 1 specimen; USNM 365517, 4; USNM 274941, 1; USNM 328251, 2; USNM 276048, 2; USNM 327608, 1.

#### Diagnosis.

A species of Starksia distinguished by the following combination of characters: orbital cirrus present; belly scaled; trunk pale (pale red in life), without distinct bars or other markings; lips peppered with white spots in life; lacrimal region with single row of small white spots in life; jaws usually with lightly scattered melanophores in preserved specimens, without distinct banding or dark bars; entire gular region usually covered with scattered melanophores; total dorsal elements usually 27; total vertebrae usually 32; dorsal spines + anal soft rays + vertebrae modally 75.

#### Description.

See Table 2. Dorsal spines XIX–XX, usually XX (XX in holotype); segmented dorsal rays 7–8, usually 8 (7); total dorsal elements 27–28, usually 27 (27); anal spines II; segmented anal rays 16–17 (16); dorsal segmented caudal-fin rays (7); ventral segmented caudal-fin rays (6); dorsal procurrent caudal-fin rays 5–6, rarely 6 (6); ventral procurrent caudal-fin rays 4–5, rarely 4 (5); obvious segmented pelvic-fin rays 2; pectoral-fin rays 12–13, rarely12 (13); vertebrae 10+21–23= 31–33, usually 32 (10+22=32); infraorbital pores usually unpaired (one pair present at 3 o’clock); orbital cirri present; nape cirri present; anterior nostril cirri present; belly and pectoral-fin base completely scaled.

**Figure 7.**
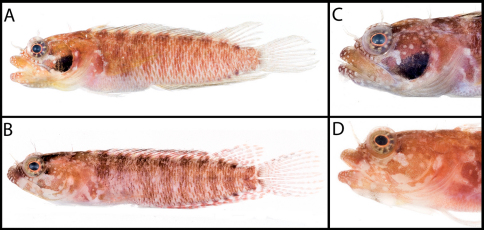
Male and female color patterns of Starksia weigti: **A** USNM 399648, holotype, BLZ 5010, 25.0 mm SL, male **B** BLZ 6121 (no voucher), 18.0 mm SL, female **C–D** close-up views of diagnostic spotting on lips in life – **C** BLZ 6120,24.0 mm SL (no voucher), male **D** USNM 399650, BLZ 5193, 24.0 mm SL, female. Photographs by Carole Baldwin and Julie Mounts.

**Table 2.** Frequency distributions of counts among species of the Starksia lepicoelia complex1.

	*Dorsal Spines*		*Dorsal Soft Rays*			*Total Dorsal*				*Anal Soft Rays*				
	XIX	XX	7	8	9	26	27	28	29	15	16	17	18	
Starksia weigti	5	11*	5*	11	-	-	10*	6	-	-	6*	10	-	
Starksia lepicoelia	2	18*	1	18*	1	-	3	16*	1	-	1	18	1*	
Starksia williamsi	10*	3	3	10*	-	-	13*	-	-	-	11*	2	-	
Starksia robertsoni	1	7*	8* -	-	1	7*	-	-	1	6*	1	-		
	*Pectoral Rays*				*Dorsal Procurrent Caudal Rays*		*Ventral Procurrent Caudal Rays*				*Vertebrae*			
	11	12	13	14	5	6	3	4	5	6	31	32	33	34
Starksia weigti	-	3	18*	-	12	2*	-	1	13*	-	1	12*	3	-
Starksia lepicoelia	-	-	18*	1	9	8	-	-	14	2	-	2	15	1
Starksia williamsi	2	1	10*	-	7	7*	-	-	13	1*	1	12*	-	-
Starksia robertsoni	-	-	6*	1	4*	3	1*	-	4	2	2*	6	-	-
	*Total Dorsal Elements + Anal Soft Rays + Vertebrae*													
	73	74	75	76	77	78	79							
Starksia weigti	-	1	5*	2	3	3	-							
Starksia lepicoelia	-	-	-	2	1	11	2							
Starksia williamsi	-	1	10*	2	-	-	-							
Starksia robertsoni	1	2*	4	1	-	-	-							

* Indicates count of holotype

1 Böhlke and Springer (1961) did not provide counts of procurrent caudal rays or vertebrae for the holotype of Starksia lepicoelia

Specimens examined ranging from 9.5 mm to 24.0 mm SL; HL 30–36% SL; length of male genital papilla two-thirds to equal length of first anal spine, papilla 1.0–1.8 mm and free from spine.

#### Pigment.

Both males and females with pale red to reddish brown trunk; indistinct vertical bars, if present, more prominent dorsally; two small (less than half pupil diameter) dark spots on posterior portion of trunk, one at posterior end of dorsal fin and one at posterior base of anal fin. Both sexes with pale red heads, scattered small white spots on anterior portions of lips, and single row of white spots beneath eye on lacrimal region; white spots representing absence of chromatophores in areas otherwise covered with pale orange to red pigment; eye with six or seven white spots around pupil, spots separated by darker areas (effectively a candy-stripe pattern). Males with prominent dark blotch on cheek and with small white spots extending from anterior portions of lips to posterior portions of jaws; females without dark cheek blotch and usually with larger white spots, blotches, or bands on posterior portions of jaws. Males with red pigment on dorsal fin confined to blotches at base and little red pigment on rest of fin and other median fins (but with scattered melanophores on dorsal, caudal, and anal fins); females with red pigment extending onto entire dorsal fin and with prominent orange/red pigment on caudal and anal fins (but without prominent melanophores); males with yellowish brown pectoral fin, females with pale orange to orange pectoral fin; pelvic fin clear.

Juvenile (BLZ 8123) color pattern: trunk pale orange, with some yellow mixed in; head with dark bar from anterior portion of eye to upper and lower lips; black triangle of pigment beneath eye; and black cap of pigment on head that extends anteriorly to vertical through middle of eye. Dorsal, anal, and caudal fins pale orange; bases of several dorsal-fin elements with darker blotches of orange pigment; most anal-fin elements with melanophore at base (typical of blennioid larvae), bases of about half of anal-fin elements also with prominent orange spot.

#### Color in preservative.

Males mostly pale, except with very dark blotch on cheek; trunk, belly, jaws, gular region, branchiostegals, operculum, top of head, nape, and all fins except pelvics with scattered melanophores, pigment on trunk fairly heavy in one male. Some females very pale, with only a few melanophores on gular region, cheek, branchiostegals, and on all fins except pelvics; other females with poorly formed dark blotch on cheek, fairly heavy pigment on gular region, branchiostegals, belly, dorsal fin, and anal fin; and lightly scattered melanophores on trunk, jaws, operculum, top of head, nape, caudal fin, and pectoral fin; pigment on head and nape usually lighter in females than in males.

Only anterior portion of body remains in juvenile voucher specimen (BZE 8123): body mostly pale; black cap of pigment on head, dark bar from anterior portion of eye to upper and lower lips, and black triangle of pigment beneath eye present in preservative.

#### Etymology.

The species name is in honor of Lee A. Weigt, Head of the Smithsonian’s Laboratories of Analytical Biology, in recognition of his contributions to the DNA barcoding of fishes and his contributions to fish-collecting efforts in Belize, Curacao, Florida, Tobago, and Turks & Caicos Islands.

#### Distribution.

Known only from Belize, Central America.

### Starksia williamsi

Baldwin & Castillo sp. n.

urn:lsid:zoobank.org:act:7C75F463-D33D-4411-8222-BAA0556FDEC4

Figs 1 8 10 11 Table 2 

#### Type Locality:

Saba Bank, Netherland Antilles

#### Holotype.

USNM 387675, sta. SABA-06-12, 21 mmSL, male, Saba Bank (Netherland Antilles), 19 m, 17°14'23"N, 63°26'55"W, 8 Jan 2006, Saba 2006 expedition team.

#### Paratypes (all Saba Bank, Netherland Antilles).

All paratypes are non-DNA vouchers except USNM 397396. USNM 397396, sta. SABA-06-01, female, just south of Poison Bank, 17°28.47'N, 63°13.40'W, 24–27 m, 4 Jan 2006 (DNA voucher of SAB 0601010—length unknown, posterior portion of body removed for DNA tissue sample); USNM 399613, sta. SABA-06-12, 3 specimens: (1) 21.5 mm SL male, (1) 22.5 mm SL female, (1) 20.0 mm SL female, 19 m, 17°14'23"N, 63°26'55"W, 8 Jan 2006;USNM 387869, sta. SABA-06-05, 4 specimens: (1) 21.5 mm SL male, (1) 19.5 mm SL male, (1) 19.5 mm SL female, (1) 19 mm SL female, overall bank, east side, 26–28 m, 17°24'36"N, 63°11'45"W, 6 Jan 2006; USNM 388033, sta. SABA-06-25, 8 specimens: (1) 22.5 mm SL male, (1) 20.5 mm SL female (1) 20.0 mm SL female (1) 19.5 mm SL female, (1) 21.5 mm SL male, (3) juveniles 8.5 -11.5 mm SL, near Coral Garden at southeast, 15–18 m, 17°21'10"N, 63°15'08"W, 14 Jan 2006. USNM 388444, sta. SABA-06-21, 4 specimens: (1)18.5 mm SL female, (3) juveniles 7.5–9.0 mm SL, northeastern shallow flats, 20 m, 17°28'03"N, 63°14'59"W, 12 Jan 2006; USNM 387767, (3) females 19.5–20.0 mm SL, (4) juveniles 8.0–11.0 mm SL, sta. SABA-06-01, just south of Poison Bank, groove in reef with sand bottom, 24–27 m, 17°28'47"N, 63°13'40"W, 4 Jan 2006.

#### Additional Material (not DNA vouchers, all Saba Bank, Netherland Antilles).

USNM 388392, 6 specimens; USNM 388589, 3; USNM 387623, 1; USNM 387733, 4; USNM 388355, 2.

#### Diagnosis.

A species of Starksia distinguished by the following combination of characters: orbital cirrus present; belly scaled; trunk pale to tan (dark orange/tan to bright orange in life), without distinct bars or other markings; lips without conspicuous white spotting, distinct banding, or dark bars—usually with lightly scattered melanophores in preserved specimens; total dorsal elements 27; total vertebrae usually 32; dorsal spines + anal soft rays + vertebrae modally 75.

#### Description.

See Table 2. Dorsal spines XIX–XX, rarely XX (XIX in holotype); segmented dorsal rays 7–8, usually 8 (8); total dorsal elements (27); anal spines II; segmented anal rays 16–17, rarely 17 (16); dorsal segmented caudal-fin rays (7); ventral segmented caudal-fin rays (6); dorsal procurrent caudal-fin rays bimodal at 5–6 (6); ventral procurrent caudal-fin rays 5–6, rarely 6 (6); segmented pelvic-fin rays 2; pectoral-fin rays 11–13, usually 13 (13); vertebrae 9–10+22= 31–32, rarely 31 (10+22=32); usually one pair of infraorbital pores at 3 o’clock (one specimen with all infraorbital pores unpaired); orbital cirri present; nape cirri present; anterior nostril cirri present; belly and pectoral-fin base completely scaled.

**Figure 8.**
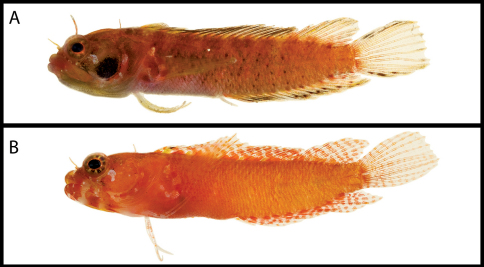
Male and female color patterns of Starksia williamsi: **A** USNM 387869, 19.5 mm SL, male, paratype **B** USNM 387767, 20.2 mm SL, female, paratype. Photographs by Jeffrey Williams.

Specimens examined ranging from 18.5 mm to 22.5 mm SL; HL 34–38% SL; male genital-papilla length between two-thirds and three-fourths length of first anal spine, papilla 1.0–1.25 mm and free from spine.

#### Pigment.

Trunk dark orange/tan to bright orange, color nearly uniform—i.e., without indistinct dark bars and pale areas; two small (less than half pupil diameter) dark spots on posterior portion of trunk, one at posterior end of dorsal fin and one at posterior base of anal fin. Both sexes with orange heads, a few small pale spots on lips and lacrimal region, and six or seven white spots around pupil, spots separated by darker areas (effectively a candy-stripe pattern). Males with prominent dark blotch on cheek and uniformly orange/tan lips; females without dark blotch on cheek and with mottling of orange and pale blotches on lips. Males with red pigment on dorsal fin largely confined to blotches at base and little red pigment on rest of fin and other median fins (but with numerous melanophores on dorsal, caudal, and anal fins); females with bright orange spotting on dorsal, anal, and caudal fins (but without prominent melanophores except one dark spot sometimes present in anterior portion of spinous dorsal); males with yellowish brown pectoral fin, females with orange pectoral fin; pelvic fin clear.

#### Color in preservative.

Males tan, usually with fairly heavy pigment on head, trunk, and dorsal-, anal-, outer caudal-, and posterior portions of pectoral-fin rays; prominent dark blotch on cheek retained in preservative; no dark spots, streaks or bars on lips. Females mostly pale, sometimes with noticeable concentrations of melanophores on cheek, jaws and gular region, but no prominent dark cheek blotch; lightly scattered melanophores usually present on branchiostegals, opercle, belly, median and pectoral fins; no conspicuous pattern of dark and pale blotches on lips, but light bar present across lips just posterior to symphysis and sometimes a few spots present just anterior to end of upper and lower jaws; posterior tips of upper and lower jaws usually pale.

#### Etymology.

Named in honor of Jeffrey T. Williams, Smithsonian’s National Museum of Natural History, in recognition of his work on blennioid fishes, including Starksia. Jeff’s field-collecting efforts at Saba Bank, Tobago, and Turks and Caicos resulted in numerous specimens utilized in this study.

#### Distribution.

Known only from Saba Bank, Netherland Antilles.

### Starksia robertsoni

Baldwin, Victor & Castillo sp. n.

urn:lsid:zoobank.org:act:2C91C572-A7FA-4BE3-BC50-C735089B018C

Figs 1 9 [Fig F10] 11 Table 2 

#### Type Locality:

Panama, Central America

#### Holotype.

AMNH 249667, 22.0 mm female, sta. JVT-07-725, Islas de Las Dos Hermanas, Portobelo, Panama, 9°35'45"N, 79°40'05"W, 2 June 2007, J. Van Tassell, D. R. Robertson, L. Tornabene, B. Victor, E. Pena (not a DNA voucher).

#### Paratypes (all from Panama).

USNM 399909, 21.0 mm SL male, PAN 1419, Islas de Las Dos Hermanas, Portobelo, 9.59577N, 79.66801W, 2 Jun 2007 ; USNM 399910, 22.0 mm SL female (not a DNA voucher), same collection information as above; USNM 399911, 20.0 mm SL male (PAN 1418), USNM 399912, 16.0 mm SL immature (PAN 014), Salmedina Reef, Portobelo, 9.56289N, 79.69557W, 31 May 2007; USNM 399913, 18.0 mm SL male (not a DNA voucher), same collection information as above; AMNH 249640, 18.0 mm SL female, sta. JVT-07-710, Salmedina Reef, Portobelo, 9°33'54"N, 79°41'54"W, 30 May 2007 (not a DNA voucher); AMNH 249642, 21.5 mm SL female, sta. JVT-07-714, Salmedina Reef, Portobelo, 9°33'46"N, 79°41'44"W, 31 May 2007.

#### Diagnosis.

A species of Starksia distinguished by the following combination of characters: orbital cirrus present; belly scaled; trunk pale to dark tan (dark orange/tan to bright orange in life), without distinct bars or other markings; lips without conspicuous white spotting in life; ventral surface of lower jaw of males with one to three dark blotches or bars in preserved specimens, lips without distinct banding or dark bars; dorsal-fin elements usually XX,7 – 27 total; vertebrae usually 10+22=32; dorsal spines + anal soft rays + vertebrae modally 75.

#### Description.

See Table 2. Dorsal spines XIX–XX, usually XX (XX in holotype); segmented dorsal rays 7; total dorsal elements 26–27, usually 27 (27); anal spines II; segmented anal rays 15–17, usually 16 (16); dorsal segmented caudal-fin rays 7; ventral segmented caudal-fin rays 6; dorsal procurrent caudal-fin rays 5–6 (5); ventral procurrent caudal-fin rays 3–6 (3); segmented pelvic-fin rays 2; pectoral-fin rays 13–14, usually 13 (13); vertebrae 10+21–22=31 or 32, usually 10+22=32 (10+21=31); infraorbital series with one pair of pores at 3 o’clock; orbital, nape, and anterior-nostril cirri present; belly and pectoral-fin base completely scaled.

**Figure 9.**
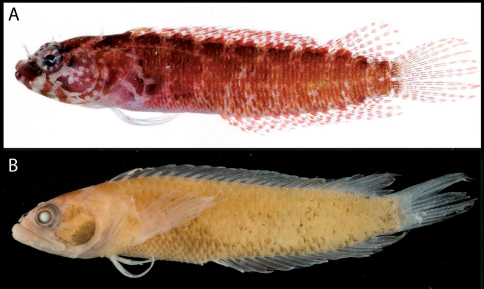
Color and preserved pigment patterns in Starksia robertsoni: **A** AMNH 249667, 22.0 mm SL, female, holotype (photograph by James Van Tassell and Ross Robertson) **B** USNM 399911, PAN 1418, 20.0 mm SL, male, paratype (photograph by Carole Baldwin).

Specimens examined ranging from 16.0–22.0 mm SL; HL 32–36% SL (32); male genital-papilla length between one-half and three-fourths length of first anal spine, papilla 0.6–1.9 mm and free from spine.

#### Pigment.

Color in life known only for two females. Trunk dark orange/tan to bright orange, color nearly uniform or with indistinct dark bars and pale areas; two small (less than half pupil diameter), inconspicuous dark spots on posterior portion of trunk, one at posterior end of dorsal fin and one at posterior base of anal fin. Head orange, mottled with white patches; a few small, pale spots present on lips and lacrimal region; eye with six or seven white spots around pupil, spots separated by darker areas (effectively a candy-stripe pattern). Bright orange spotting on dorsal, anal, and caudal fins, and some orange pigment on pectoral fin; pelvic fin clear.

#### Color in preservative.

Trunk ranging from pale to dusky, belly with fairly heavy pigment in males and some females even if trunk pale. Males usually with prominent dark blotch on cheek (largest male, USNM 399909, PAN1419, with dark spots on cheek but no conspicuous blotch), females without dark cheek blotch. Underside of lower jaw with one to three dark spots or bars in males, middle one (situated roughly beneath a vertical through pupil) darkest and sometimes the only one noticeable; anterior marking, if present, sometimes extending onto lower lip as a few dark dots; no dark spots, streaks, or bars on lips in either sex, but portions of lips uniformly covered with melanophores in males and with at least a few spots in females; females usually with patch or bar of pigment (small and faint in some specimens) extending from lacrimal region across both lips. In males, branchiostegals dusky, upper part of cheek, opercle, and top of head pale to dusky; in females, head mostly pale, with isolated patches of spots on cheek, opercle, top of head, and branchiostegals. Dorsal, anal, caudal, and pectoral fins dusky in males, mostly pale in females with a few scattered spots on some fins.

#### Etymology.

Named in recognition of the contributions by D. Ross Robertson of the Smithsonian Tropical Research Institute to the understanding of the diversity of shorefishes of the New World and his generous facilitation of collecting in Panama.

#### Distribution.

Known only from Panama (Atlantic)

### Comparisons among Species of the Starksia lepicoelia Complex (Figs 10–11)

**Comparative material.** Starksia lepicoelia. Bahamas (DNA vouchers): USNM 399615, BAH 8077; USNM 399616, BAH 8078; USNM 399617, BAH 8079. Bahamas (not DNA vouchers): USNM 399923, 1 specimen; USNM 399924, 1; USNM 399925, 1; USNM 399926, 1; USNM 399927, 9; USNM 399928, 1; USNM 399929, 1; USNM 399930, 1; USNM 399931, 1; USNM 399932, 1; USNM 399933, 1; USNM 399934, 1; USNM 399921, 1; USNM 399922, 1; USNM 386919, 3 specimens; USNM 386972, 15; USNM 386383, 1; USNM 386402, 8; USNM 386651, 2; USNM 386581, 3; USNM 386500, 4; USNM 387026, 3; USNM 386244, 13; USNM 387069, 6; USNM 399618, 1; USNM 399614, 2; Turks and Caicos Islands (DNA vouchers): USNM 399638, TCI 9291; USNM 399639, TCI 9292; USNM 399640, TCI 9293; USNM 399641, TCI 9294; USNM 399636, TCI 9112; Turks and Caicos Islands (not DNA vouchers): USNM 399637, 7; USNM 399642, 1. Navassa Island (not DNA vouchers): USNM 359448, 5; USNM 359699, 19.U.S. Virgin Islands, St. Croix (not DNA vouchers): UF 149809, 11; UF 149815, 33; UF 149814, 10.

**Figure 10. F10:**
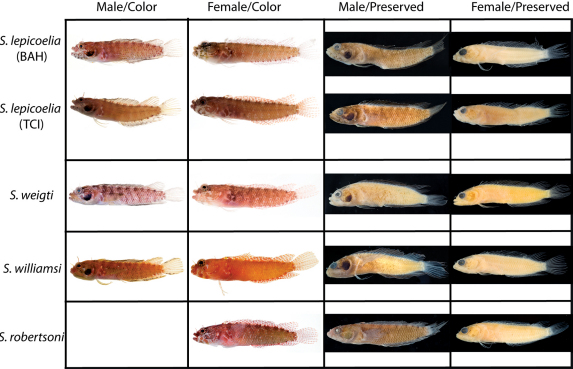
Comparisons among species of the Starksia lepicoelia complex. Left to right: Starksia lepicoelia (BAH): USNM 399928, BAH 10050, 25.0 mm SL; USNM 399617, BAH 8079, 19.0 mm SL; USNM 399921, BAH 9103, 26.0 mm SL; USNM 386972, 14.0 mm SL; Starksia lepicoelia (TCI): USNM 399638, TCI 9291, 23.5 mm SL; USNM 399641, TCI 9294, 25.5 mm SL; USNM 399642, 23.0 mm SL; USNM 399641, TCI 9294, 25.5 mm SL; Starksia weigti: BLZ 6120, 24.0 mm SL (no voucher); USNM 399650, BLZ 5193, 24.0 mm SL; USNM 399648, BLZ 5010, holotype, 20.5 mm SL; USNM 274922, paratype, 20.0 m SL; Starksia williamsi: USNM 387767, 19.8 mm SL; USNM 387767, 20.2 mm SL; USNM 387675, holotype, 21.0 mm SL; USNM 387869, paratype, 19.5 mm SL; Starksia robertsoni: AMNH 249642, paratype, 21.5 mm SL; USNM 399909, PAN 1419, paratype, 21.0 mm SL; AMNH 249667, holotype, 22.0 mm SL. Photographs by Carole Baldwin, Cristina Castillo, Donald Griswold, Ross Robertson, James Van Tassell, and Jeffrey Williams.

**Figure 11.**
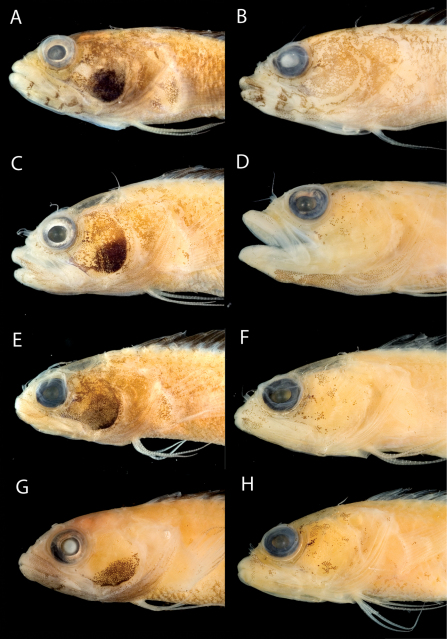
**A** Comparisons of head pigment of preserved males and females among species of the Starksia lepicoelia complex. Starksia lepicoelia: **A** USNM 399921, BAH 9103, 26.0 mm SL, male **B** USNM 399617, BAH 8079, 19.0 mm SL, female; Starksia weigti: **C** USNM 399648, BLZ 5010, holotype, 20.5 mm SL, male **D** USNM 399651, BLZ 8024, paratype, 19.0 mm SL, female; Starksia williamsi: **E** USNM 387675, holotype, 21.0 mm SL, male **F** USNM 387869, paratype, 19.5 mm SL, female; Starksia robertsoni: **G** USNM 399913, paratype, 18.0 mm SL, male **H** AMNH 249667, holotype, 22.0 mm SL, female. Photographs by Carole Baldwin, Cristina Castillo, and Donald Griswold.

#### Comparisons.

Starksia lepicoelia and Starksia starcki are the only previously described western Atlantic Starksia with the combination of an orbital cirrus, two externally obvious pelvic-fin rays, and a scaled belly (Williams and Mounts 2003). Starksia starcki is easily distinguished from the species of the Starksia lepicoelia complex by the presence of eight or nine irregular dark bars on the body and usually 19 segmented anal-fin rays.

In life, Starksia weigti is easily distinguished from Starksia lepicoelia, Starksia williamsi, and Starksia robertsoni by the conspicuous pale round spots on the lips. In preservative, Starksia lepicoelia males are distinctive in having at least some very dark spots, streaks, or bars on the lips and lower jaw, and Starksia robertsoni males have at least one (up to three) dark spots or bars on the ventral portion of the lower jaw (but not on the lips). Although the differences are subtle, preserved males of Starksia williamsi typically can be separated from preserved males of Starksia weigti in having the lips uniformly covered with melanophores except for the pale anterior tips. In Starksia weigti males, lip pigment is variable, but there are usually one or two thin, faint, poorly formed bars of pigment following the pale anterior portions of the lips; posteriorly, the lips may be uniformly covered with melanophores as in Starksia williamsi or be quite pale.

Preserved female Starksia lepicoelia also have a distinctive lip pattern—alternating pale and dark areas. Although this banding pattern appears to be present in color images of Starksia williamsi, Starksia weigti, and Starksia robertsoni, it is not present in preserved females of those species, suggesting that in Starksia lepicoelia the banding comprises both chromatophores and melanophores whereas in females of the other species it comprises only chromatophores and thus is not retained in preservative. As in males, differences in head pigment between preserved female Starksia williamsi and Starksia weigti are subtle, but Starksia williamsi females have a relatively well-formed bar of pigment from the anterior portion of the lacrimal across both lips, whereas Starksia weigti females typically have only a light scattering of melanophores on the upper lip beneath the anterior portion of the lacrimal. Additionally, Starksia williamsi females tend to have a bit of dark pigment at the posteroventral corner of the orbit and another bit just ventral to posteriormost point of orbit; Starksia weigti females usually have more widely scattered pigment on the cheek -- sometimes in a fairly cohesive spot. The head pigment of female Starksia robertsoni is very similar to that of Starksia williamsi, but modal differences in fin-ray counts separate them, and they are geographically distinct. Specifically, Starksia williamsi—from the eastern Caribbean—typically has XIX,8 dorsal-fin elements, whereas Starksia robertsoni—from Panama—typically has XX,7.

Modal differences in some counts also help separate other species: Starksia lepicoelia modally has 28 total dorsal-fin elements, 33 vertebrae, and 78 total dorsal elements + anal soft rays + vertebrae (vs. 32, 27, and 75, respectively, in Starksia williamsi and Starksia weigti). Starksia williamsi modally has XIX dorsal-fin spines, whereas Starksia lepicoelia and Starksia weigti modally have XX.

We examined color photographs and numerous preserved specimens from St. Croix, U.S. Virgin Islands, but we do not have genetic data for that material. Fresh specimens lack the diagnostic white spots on the lips of Starksia weigti. Preserved specimens most closely resemble Starksia lepicoelia in pattern of pigment on the lips and lower jaw, with females typically having at least some alternating pale and dark areas (nearly identical to that of Starksia lepicoelia in some specimens, not distinctive at all in others). Although most males have fairly uniform pigment on the lips and lower jaw, at least some males have the distinctive dark bars, spots, or streaks characteristic of male Starksia lepicoelia. If the St. Croix specimens represent one of the known Starksia lepicoelia species, it seems likely based on geography and pigmentation that they are Starksia lepicoelia. However, we note that Starksia lepicoelia typically has 28 total dorsal elements and 17 anal-fin soft rays, whereas the St. Croix specimens (15 counted) typically have 27 and 16, respectively (but 28 dorsal elements and 17 anal rays are not uncommon counts). Additional investigation, including genetic analysis, is needed.

### Key to Species of the Starksia lepicoelia Complex

**Table:** 

1a	Lips with distinct dark bars or blotches in preserved males; lips and lower jaw with alternating pale and darker areas in preserved females; total vertebrae modally 33; total dorsal elements + anal soft rays + vertebrae modally 78	Starksia lepicoelia (Bahamas, Turks and Caicos)
1b	Lips without distinct dark markings in preserved males; lips and lower jaw without conspicuous alternating pale and darker areas in preserved females; total vertebrae modally 32; total dorsal elements + anal soft rays + vertebrae modally 75	2
2a	Preserved males with one to three small dark spots or bars on ventral portion of lower jaw; dorsal-fin elements modally XX,7	Starksia robertsoni Panama (Atlantic)
2b	Preserved males without dark markings on ventral portion of lower jaw; dorsal-fin elements modally XIX,8 or XX,8	3
3a	Lips with conspicuous pattern of white spotting in life; dorsal-fin spines modally XX (also see “Comparisons,” above)	Starksia weigti (Belize)
3b	Lips with few or no white spots in life; dorsal-fin spines modally XIX (also see “Comparisons,” above)	Starksia williamsi (Saba Bank, Netherland Antilles)

## Starksia sluiteri Species Complex

Metzelaar (1919) described Brannerella sluiteri from two specimens from Bonaire, Netherland Antilles. Longley (1934) synonymized Brannerella with Starksia Jordan and Evermann (type species Labrisomus cremnobates Gilbert, from the eastern Pacific). Böhlke and Springer (1961) concurred with Longley’s synonymy, noting that Brannerella is distinctive in a single character, and generic recognition of one-character differences would require the erection of several new genera within Caribbean Starksia.

Our material includes three genetic lineages originally identified as Starksia sluiteri based on the taxonomic key of Williams and Mounts (2003) — one from Curacao, one from Tobago, and one from Belize/Honduras/Panama. Specimens in all three lineages modally have 13 pectoral-fin rays, 20 or fewer dorsal-fin spines, and two or three rows of dark spots or blotches along the body — features typical of Starksia sluiteri. We have identified our genetic lineage from Curacao (CUR in Fig. 1) as Starksia sluiteri (Metzelaar) based on geography and morphology. In particular, the second row of dark markings (middle row when there are three) are distinctly round in Starksia sluiteri and in our Curacao specimens, whereas those markings are usually vertically elongate in our specimens from Belize (BLZ), Honduras (HON), and Panama (PAN). Additionally, although Metzelaar (1919) illustrated a male specimen in his original description, he did not mention any round, pale markings on the head—prominent diagnostic features in males of our specimens from Tobago (TOB) that are lacking in our male Starksia sluiteri from Curacao. We recognize the genetic lineage from Tobago, as well as that from Belize/Honduras/Panama, as new species within the Starksia sluiteri complex and provide descriptions below.

Böhlke and Springer (1961) noted that counts of dorsal- and anal-fin elements in specimens of Starksia sluiteri they examined from off Colombia and Venezuela (XIX dorsal spines and 15–16 anal rays) differ from those given by Metzelaar (XX and 17). Based on pigment, their Colombian and Venezuelan specimens appear to be Starksia sluiteri. Our specimens from Curacao, as well as Böhlke and Springer’s two Venezuelan specimens (USNM 195750), have XIX dorsal spines and 15–16 anal rays. There is thus a discrepancy between counts in our material and those reported by Metzelaar for the holotype. We examined a photograph of the holotype, and there appear to be XX dorsal-fin spines as noted by Mezelaar; XX is likely a non-modal count for Starksia sluiteri. We note that there is more variation in dorsal- and anal-fin counts in some Starksia species than suggested by Metzelaar’s description; for example, Starksia greenfieldi has XVIII–XX dorsal spines, 7–9 dorsal rays, and 14–16 anal rays.

### Starksia greenfieldi

Baldwin & Castillo sp. n.

urn:lsid:zoobank.org:act:CFD1A620-8C85-4DC3-82A9-8A86BAE66C2A

Figs 1 12 15 Table 3

Starksia sluiteri Williams and Mounts 2003, Aqua 6(4): Fig. 9 (male and female specimens from Tobago)

#### Type Locality:

Tobago, Trinidad and Tobago

#### Holotype.

USNM 320832, male, 19.0 mm SL (not a DNA voucher), sta. JTW 90-9, vertical wall just north of Charlotteville on east side of North Point, Tobago, 5–12 m, 8 Sep 1990, J. T. Williams, J. Howe, S. Blum, D. Johnson, S. Love, M. Schotte.

#### Paratypes (all from Tobago).

USNM 398919, male, 22.0 mm SL (not a DNA voucher), same locality information as for holotype; USNM 398922, TOB 9282, female, 19.0 mm SL, sta. TOB09-8, rock/coral outcrops on sand, Pirate’s Bay, Charlotteville, < 3 m, 11°19.300'N, 60°32.977'W, 18 Mar 2009 (small fillet removed from right side for DNA tissue sample). USNM 398921, TOB 9275, male, 17.0 mm SL, collected in same station, TOB09-8, as USNM 398922 above (small fillet removed from right side for DNA tissue sample); USNM 398920, TOB 9212, male, 15.0 mm SL, sta. TOB09-6, Buccoo Reef, 9–11 m, 11°11.167'N, 60°50.761'W, 17 Mar 2009 (posterior portion of body destroyed for DNA tissue sample); USNM 398924, sta. TOB09-11, 4 specimens: (1) 12.0 mm SL juvenile, (2) 18.0 mm SL females, (1) 19.5 mm SL female (not DNA vouchers), Store Bay, 5–9 m, 11°09.349'N, 60°50.535'W, 16 Mar 2009; USNM 398923, sta. TOB 09-1, (1) 17.0 mm SL male (not a DNA voucher), coral heads/coral rubble off Mt. Irvine Beach (Hotel Beach), < 1 m, 11°11.786'N, 60°47.768'W, 15 Mar 2009; USNM 320829, sta. JTW 90–11, female, 22.0 mm SL (not a DNA voucher), coral rubble/sand, Buccoo Reef (reef crest and lagoon side of reef), 1–3 m, 11°11'12"N, 60°49'30"W, 10 Sep 1990.

#### Additional Material (all Tobago).

USNM 398925, TOB 9213; USNM 398926, TOB 9214; USNM 398918, 19 specimens; USNM 398917, 16; USNM 320823, 5.

#### Diagnosis.

A species of Starksia distinguished by the following combination of characters: orbital cirrus present; two to three rows of dark blotches on side of body, blotches in middle row (or ventral row if only two rows) mostly circular, never vertically elongate or oval; white (or pale), mostly round spots (absence of melanophores against a darker background) on at least portions of cheek, opercle, and gular region, this spotting pattern more prominent in males; males with dark blotch of pigment on anterior portion of spinous dorsal fin; first anal-fin spine one-half to three-quarters length of male genital papilla; belly naked.

#### Description.

See Table 3. Dorsal spines XVIII–XX (XIX); segmented dorsal rays 7–9, modally 8 (7); total dorsal elements 26–28, modally 27 (26); anal spines (II); segmented anal rays 15–16, modally 16 (15); dorsal segmented caudal-fin rays 7; ventral segmented caudal-fin rays 6; dorsal procurrent caudal-fin rays 5–6 (5); ventral procurrent caudal-fin rays 5–6, rarely 6 (5); segmented pelvic-fin rays 2; pectoral-fin rays 13–14, rarely 14 (13); vertebrae 10+21–23=31–33, usually 10+22=32 (10+22=32); infraorbital pore arrangement variable—unpaired (condition in holotype), one pair at 3 o’clock, two pairs (3 and 6 o’clock), and one specimen with three pairs (3, 4, and 5 o’clock); orbital, nape, and anterior-nostril cirri present and; belly and pectoral-fin base completely naked.

**Figure 12.**
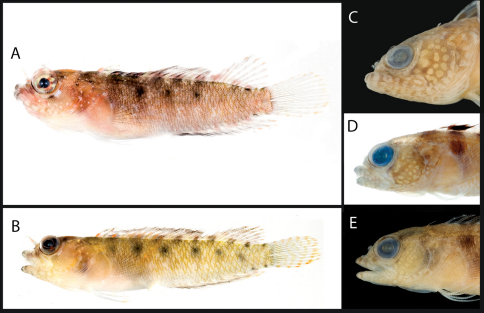
Male and female color patterns of Starksia greenfieldi:**A** USNM 398920, TOB 9212, 15.0 mm SL, male **B** USNM 398922, TOB 9282, 19.0 mm SL, female **C–E** Diagnostic features of preserved Starksia greenfieldi - **C** USNM USNM 398919, paratype, male, 22.0 m SL, note pale spots on head **D** USNM 320832, holotype, male, 19.0 mm SL, note pale spots on head and dark blotch in anterior portion of spinous dorsal fin **E** USNM 320829, female, 22.0 mm SL, note pale spots on head. Photographs by Carole Baldwin, Cristina Castillo, and Donald Griswold.

**Table 3.** Frequency distributions of counts among species of the Starksia sluiteri complex1.

	*Dorsal Spines*			*Dorsal Soft Rays*			*Total Dorsal*			*Anal Soft Rays*		
	XVIII	XIX	XX	7	8	9	26	27	28	15	16	17
Starksia greenfieldi	7	14*	1	5*	12	5	6*	15	1	6*	16	-
Starksia langi	-	10*	1	6*	4	-	5*	5	-	7*	1	-
Starksia sluiteri	-	5	1*	2*	3	1	1	4*	1	2	1	1*
	*Pectoral Rays*			*Dorsal Procurrent Caudal Rays*		*Ventral Procurrent Caudal Rays*		*Vertebrae*				
	12	13	14	5	6	5	6	31	32	33		
Starksia greenfieldi	-	24*	1	8*	10	16*	1	1	11*	2		
Starksia langi	-	12*	-	3*	4	6*	-	4*	3	-		
Starksia sluiteri	1	4	-	1	-	1	-	-	2	-		

* Indicates count of holotype

1 Metzelaar (1919) did not provide counts of pectoral-fin rays or vertebrae for the holotype of Starksia sluiteri

Specimens examined ranging from 11.0–23.0 mm SL; HL 30–36% SL (36%); length of male genital papilla 19–26% SL in specimens 19.0 mm SL and larger (26%), 12–14% in specimens 15.0–17.0 mm SL; papilla adhered to first anal-fin spine and extending well beyond it, spine one-half length of papilla in most males, greater than three-quarters in smallest males.

#### Pigment.

Head and body pale yellow to pale orange, generally more orange in males, more yellow in females; posterior margins of most body scales covered with yellow or orange chromatophores mixed with melanophores, resulting in background pattern of chain-link or diamond-shaped markings. Two or three rows of dark markings on trunk in mature specimens, markings diffuse in some specimens: dorsalmost row with 7–10 roughly square blotches that extend onto bases of dorsal-fin elements (another dark blotch on nape in line with this row of markings); second row with 6–7 circular blotches situated just above lateral midline; lower row, if present, with 1–4 diffuse, round to oblong blotches. A few to many white, mostly round spots on at least portions of cheek, opercle, and gular region and sometimes lower jaw; this pattern resulting from the absence of melanophores against a darker background and typically significantly more prominent in males. Males also differing from females in having dark blotch of pigment on anterior portion of spinous dorsal fin. Distinctive, dark-orange markings usually present on proximal portion of dorsal fin where dark blotches in dorsalmost row of markings on body extend onto dorsal fin; where those dark blotches extend onto two (vs. one) dorsal-fin element, dark orange markings distinctly paired. Orange pigment also present on distal portions of pectoral-fin rays and lighter orange pigment present on at least distal portions of second dorsal-, caudal-, and posterior anal-fin rays; sometimes orange blotches present intermittently along lengths of second dorsal-, caudal-, and anal-fin rays forming wavy stripes or bars of pigment on those fins. Orange pigment present on top of head, in bright ring around eye, and on nasal cirrus. Some specimens with dark orange pigment on snout, in blotches radiating from pupil, on operculum, and on dorsal portions of pectoral-fin base. In one specimen most chromatophores on head and body yellow to yellowish orange, but those on nasal cirrus, around eye, and on fins distinctly orange.

#### Color in preservative.

Diagnostic dark markings on trunk present as described above; diagnostic white, round spots on head described above present as distinctive pale markings in preserved specimens—head markings especially prominent in large males; trunk largely tan and peppered with dark dots, especially along posterior margins of scales; lips with mottled or barred pigment pattern; a fairly uniform covering of melanophores on snout, branchiostegals, pectoral-fin base, and belly; eye sometimes surrounded by dark ring of pigment; top of head and nape usually darker than rest of head, pigment on nape usually in form of dark saddle extending over dorsal midline; two concentrations of melanophores usually visible on brain; dorsal and anal fins dusky, dark body blotches in upper row usually extending onto base of dorsal fin; dorsal fin of males with dark blotch between spines II–IV; caudal-fin rays edged with dark pigment, outer rays with more uniform scattering of melanophores; proximal portion of pectoral fin covered with scattered melanophores, distal portion with dark edging along rays; males sometimes with pigment on membranes between some pectoral rays distally; pelvic fin clear.

#### Etymology.

The species name is in honor of David W. Greenfield, in recognition of his work on blennioid fishes, particularly his work on the Starksia ocellata complex.

#### Distribution.

Known only from Tobago

### Sarksia langi

Baldwin & Castillo sp. n.

urn:lsid:zoobank.org:act:3C78FE0F-BFD6-4F14-9E91-4DD3825A67AE

**Figure 13.**
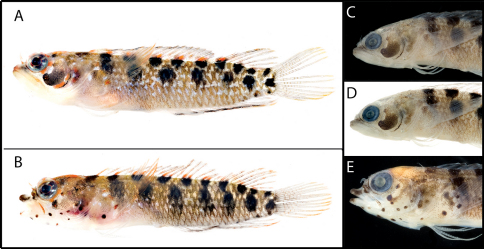
Male and female color patterns of Starksia langi:**A** USNM 398931, paratype, BLZ 8266, 18.0 mm SL, male **B** USNM 398929, paratype, BLZ 8131, 16.0 mm SL, female **C–E** Diagnostic features of preserved Starksia langi –(**C** and **D**) USNM 398931, paratype, BLZ 8266, male, 18.0 mm SL, note dark marking on cheek and absence of dark blotch in anterior portion of spinous dorsal fin **E** USNM 398928, paratype, BLZ 8062, female, 17.0 mm SL, note small dark spots on head. Photographs by Carole Baldwin, Cristina Castillo, and Donald Griswold.

Figs 13 [Fig F14] 15 Table 3 

#### Type Locality:

Belize, Central America

#### Holotype.

USNM 398927, female, 17.0 mm SL (not a DNA voucher), sta. CB08-19, inside and outside of Curlew Reef, Belize, 0–3 m, 21 May 2008, C. Baldwin and Z. Foltz.

#### Paratypes.

USNM 398928, BLZ 8062, female, 17.0 mm SL, sta. CB08-5, patch reef at south end of Carrie Bow Cay, Belize, 0–3 m, 16 May 2008 (posterior portion of body removed for DNA tissue sample). USNM 398929, BLZ 8131, female, 16.0 mm SL, sta. CB08-11, coral heads on sand bottom, Glover’s Reef, Belize, 0–3 m, 16°43'08.4"N, 87°53'13.1"W, 18 May 2008 (posterior portion of body removed for DNA tissue sample); USNM 398930, BLZ 8216, female, 11.5 mm SL, sta. CB08-20, south end of Carrie Bow Cay, Belize, 0–3 m, 21 May 2008 (posterior portion of body destroyed for DNA tissue sample); USNM 398931, BLZ 8266, male, 18.0 mm SL, sta. CB08-27, south end of Carrie Bow Cay, 0- m, 23 May 2008 (posterior portion of body removed for DNA tissue sample); USNM 349080, male, 18.0 mm SL (not a DNA voucher), reef crest in front of Carrie Bow Cay, Belize, 16 July 1991; USNM 399917, HON 050, male, 16.3mm SL, Utila, Bay Islands, Honduras, 3 Jul 2008.

#### Additional Material.

Belize: USNM 317476, 1 specimen (not a DNA voucher). Colombia (Cayos del Este): UF 223370, 5 (not DNA vouchers)—counts made from 1 male and 1 female, both 16.0 mm SL included in Table 3. Colombia (Isla Providencia): MZUSP 107860, 1 (not a DNA voucher). Panama (San Blas Islands): USNM 399918, PAN 018.

#### Diagnosis.

A species of Starksia distinguished by the following combination of characters: orbital cirrus present; two rows of prominent, very dark blotches on side of body, at least some of those in lower row vertically elongate to oval, rarely round; males with dark, fat, crescent-shaped marking on cheek and without dark blotch on anterior portion of spinous dorsal fin; females with scattered dark spots on lower half of head and on pectoral-fin base; first anal-fin spine in males two-thirds to three-quarters length of male genital papilla; belly naked.

#### Description.

See Table 3. Dorsal spines XIX–XX, rarely XX (XIX); segmented dorsal rays 7–8 (7); total dorsal elements bimodal at 26–27 (26); anal spines II; segmented anal rays 15–16, rarely 16 (15); dorsal segmented caudal-fin rays 7; ventral segmented caudal-fin rays 6; dorsal procurrent caudal-fin rays 5–6 (5); ventral procurrent caudal-fin rays 5; segmented pelvic-fin rays 2; pectoral-fin rays 13; vertebrae 10+21=31,10+22=32, or 11+21=32 (10+21=31); infraorbital pore arrangement variable—unpaired (condition in holotype), one pair at 3 o’clock, or two pairs (3 and 4 o’clock); orbital, nape, and anterior-nostril cirri present; belly and pectoral-fin base completely naked.

Specimens examined ranging from 9.0–19.0 mm SL; HL 29–33% SL (29%); length of male genital papilla 19–22% SL; papilla adhered to first anal-fin spine and extending well beyond it, spine two-thirds to three-quarters length of papilla.

#### Pigment.

Head and body pale orange; posterior margins of most body scales covered with yellow or orange chromatophores mixed with melanophores, resulting in background pattern of chain-link or diamond-shaped markings. Two rows of dark markings on trunk: dorsal row with 9 roughly circular blotches that extend onto bases of dorsal-fin elements (another dark blotch on nape in line with this row of markings); ventral row with 6–7 blotches along middle of trunk, at least some vertically elongate to oval in shape; blotches generally not round, although one or more within row may be roughly so. Females with small dark spots on cheek, operculum, branchiostegals, lower jaw, gular, and pectoral-fin base; spots smaller than pupil (several would fit in pupil) but much larger than tiny dark dots that pepper most of head and trunk; males with dark, fat, crescent-shaped marking on cheek; orange chromatophores associated with head markings in both sexes. Both males and females lacking dark blotch of pigment on anterior portion of spinous-dorsal fin. Prominent orange markings present on bases of dorsal-fin elements above dark blotches along dorsal portion of trunk; where dark blotches extend onto bases of two dorsal-fin elements, orange markings distinctively paired; other orange pigment including chromatophores on top of head, around eye, on nasal cirrus, and on tips of pectoral-, dorsal-, caudal-, and anal-fin rays; those on pectoral fin bright orange.

#### Color in preservative.

Diagnostic dark blotches on trunk present as described above; diagnostic small dark spots on head in females and large blotch on cheek in males also distinctive in preserved specimens; body overall tan to dark tan. Males with uniform scattering of spots on lips and rest of head and pectoral-fin base; dorsal, caudal, anal, and pectoral rays dusky -- i.e., with pigment on membranes between fin rays. Females with dark spots on lips, chin, snout, circumorbitals, and pectoral-fin base; top of head and nape densely covered with melanophores; dorsal, caudal, anal, and pectoral rays edged in dark spots, but little or no pigment on membranes between fin rays. Dark blotches on dorsal portion of trunk extending onto dorsal-fin rays in both sexes; belly pale to lightly pigmented; pelvic fin clear.

#### Etymology.

Named in honor of Michael A. Lang, Director of the Smithsonian Marine Science Network (MSN) and Smithsonian Science Diving Program, in gratitude for the support MSN has provided for our Caribbean fish diversity studies and in recognition of the contributions Michael has made to science diving.

#### Distribution.

Known from Belize, “Colombia,” Honduras, and Panama (see “Remarks” below).

#### Remarks.

**Figure 14. F14:**
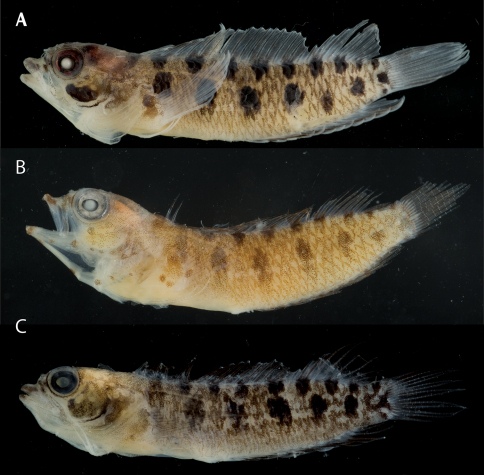
Starksia langi. **A** Male from Honduras, USNM 399917, HON 050, paratype, 16.3 mm SL (right side, reversed) **B** Femalefrom Panama (Atlantic), USNM 399918, PAN 018, 14.5 mm SL **C** Male from Isla Providencia, Colombia, MZUSP 107860, 16 mm SL. Photographs by Carole Baldwin.

A tissue sample from a single specimen off Honduras (HON 050 on tree in Fig. 1) produced a COl sequence very similar to those of our Belize specimens, and one from Panama (PAN 018) is approximately 1% different. The Honduras specimen (Fig. 14A) has the diagnostic pigment on the cheek of male Starksia langi, and the Panama specimen (Fig. 14B) has the diagnostic small dark dots of female Starksia langi. We recognize the Honduras and Panama specimens as Starksia langi.

We lack tissue samples of Colombian specimens, but the five specimens in UF 223370 from Cayos del Este (San Andrés) and a 16-mm SL specimen from Isla Providencia (Fig. 14C) appear to have the vertically elongate pigment blotches on the trunk diagnostic of Starksia langi. Pigment is somewhat faded in the UF specimens, but the 16-mm SL female in the lot has dark spots on the head as in female Starksia langi. Although we include “Colombia” in the distribution list of this species above, we note that the Colombian specimens are from the Archipelago of San Andrés, Providencia, and Santa Catalina, a group of islands nearly 800 km from Colombia but only 220 km from Nicaragua. We have no material from continental Colombia, but Starksia sluiteri replaces Starksia langi off Venezuela.

### Comparisons among Species of the Starksia sluiteri Complex (Fig. 15)

**Comparative material.** Starksia sluiteri. Curacao (all DNA vouchers): USNM 399623, CUR 8162; USNM 399624, CUR 8226; USNM 399625, CUR 8227; USNM 399626, CUR 8271. Los Roques, Venezuela (not DNA vouchers): USNM 195750, 2 specimens. Dominica (not DNA vouchers): USNM 198263, 15. Puerto Rico (not a DNA voucher): USNM 219143, 1. Antigua (not a DNA voucher): UF 11344, 1. Mexico (not DNA vouchers): UF 209342, 2. Starksia fasciata, Turks & Caicos Islands (all DNA vouchers): USNM 399681, TCI9204; USNM 399683, TCI 9349; USNM 399684, TCI 9350; USNM 399685, TCI 9714. Starksia sp. Navassa Island (not DNA vouchers): USNM 361059, 2.

**Figure 15.**
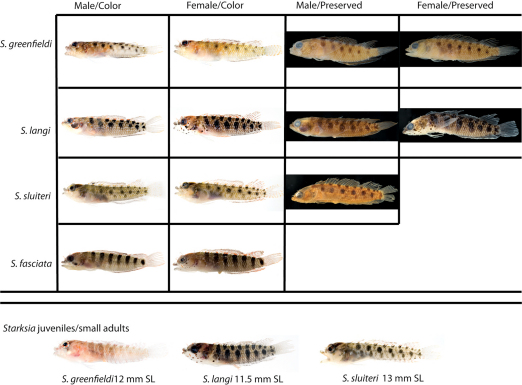
Comparisons among species of the Starksia sluiteri complex and Starksia fasciata. Starksia greenfieldi, left to right: USNM 398921, paratype, TOB 9275, 17.0 mm SL; USNM 398922, paratype, TOB 9282, 19.0 mm SL; USNM, 320832, holotype, 19.0 mm SL; USNM 320829, paratype, 22.0 mm SL. Starksia langi: USNM 398931, BLZ 8266, 18.0 mm SL; USNM 398928, BLZ 8062, 17.0 mm SL; USNM 349080, paratype, 18.0 mm SL; USNM 398927, holotype, 17.0 mm SL. Starksia sluiteri: USNM 399626, CUR8271, 16.5 mm SL; USNM 399624, CUR8226, 18.5 mm SL; USNM 195750, 16.9 mm SL. Starksia fasciata:USNM 399681, TCI 9204, 14.0 mm SL; USNM 399683, TCI 9349, 18.0 mm SL. Juveniles/small adults: Starksia greenfieldi, USNM 398925, TOB 9213; Starksia langi, USNM 398930, paratype, BLZ 8216; Starksia sluiteri, USNM 399625, CUR 8227. Photographs by Carole Baldwin, Cristina Castillo, Donald Griswold, and Jeffrey Williams.

Starksia langi is easily distinguished from Starksia greenfieldi and Starksia sluiteri based on pigmentation of the trunk, head (females), and first dorsal fin (males). The trunk pigment of Starksia langi comprises both larger and more prominent markings than that of Starksia greenfieldi and Starksia sluiteri, and only in Starksia langi are the markings in the second row vertically elongate (generally round in the other species and sometimes considerably more diffuse in Starksia greenfieldi). Starksia greenfieldi lacks dark markings on the head in both sexes, and Starksia sluiteri lacks them in females; Starksia langi males have a prominent dark blotch on the cheek, and females have numerous small, discrete, dark spots. Males of Starksia langi lack a dark blotch on the anterior portion of the dorsal fin, whereas this blotch is present in Starksia greenfieldi and Starksia sluiteri.

Starksia greenfieldi can be distinguished from Starksia langi and Starksia sluiteri by the white (or pale), mostly round spots (absence of melanophores against a darker background) on at least portions of cheek, opercle, and gular region. This pattern is present in both sexes but is often much more prominent in males. Williams and Mounts (2003) noted that Starksia sella, another species of Starksia known only from Tobago, has small pale spots on the head, but that species lacks dark blotches along the trunk, lacks a dark blotch in the anterior dorsal fin of males, and may be larger (Williams and Mounts specimens of Starksia sella are 13.7–27.7 mm SL, our specimens of Starksia greenfieldi are 11.0–23.0 mm SL).

Starksia sluiteri (Metzelaar) is most easily distinguished from Starksia langi by having the second row of trunk blotches almost perfectly round (vs. vertically elongate), in lacking conspicuous dark spots on the head (females), and in having a dark marking on the anterior portion of the dorsal fin (males). From Starksia greenfieldi, Starksia sluiteri differs in lacking pale round spots on the head. Although Starksia sluiteri and Starksia langi have very similar chromatophore patterns, Starksia sluiteri appears to have more orange pigment on the second dorsal, caudal, and anal fins.

In their descriptions of Starksia leucovitta, Starksia melasma, Starksia multilepis, Starksia rava, and Starksia sella, Williams and Mounts (2003) noted that those species belong to the Starksia sluiteri complex. Large genetic distances separate the species of the Starksia sluiteri complex, and our Starksia multilepis samples from Brazil are nearly as similar genetically to Starksia sluiteri as Starksia langi is (Fig. 1). We have no tissue samples of the other proposed members of the Starksia sluiteri complex for comparative purposes. Those species are not very similar to Starksia sluiteri in trunk pigment, particularly in lacking any bold markings. Starksia fasciata from the Turks and Caicos Islands (TCI 9204, TCI 9349, TCI 9350) is embedded within our Starksia sluiteri complex (Fig. 1), and Starksia fasciata is morphologically similar to species in that complex (Fig. 15). In Williams and Mounts (2003) diagnostic key, Starksia fasciata and Starksia sluiteri are in the same couplet, separated by pattern of pigment on the trunk (bars of trunk pigment in the former, rows of dark blotches in the latter). Male and female Starksia fasciata from the Turks and Caicos Islands (Fig. 15) are very similar to male and female Starksia langi from Belize in head pigmentation and in having prominent orange markings along the base of the dorsal fin. More material is needed to determine if Starksia smithvanizi, a species that Williams and Mounts (2003) considered part of the Starksia fasciata complex, also is genetically aligned with the Starksia sluiteri complex. We reiterate that our neighbor-joining tree (Fig. 1) is not intended to reflect phylogenetic relationships, and a species-level phylogeny derived from multiple genes should help resolve species and supra-specific relationships in the Starksia sluiteri complex.

Museum specimens examined from the Lesser Antilles (Dominica) and Puerto Rico appear to be Starksia sluiteri based on trunk pigment (round vs. elongate blotches in the second row of markings) and no conspicuous round pale spots on the cheek. The pigment is somewhat faded in those specimens, however, and more material, including tissue samples for genetic analysis, is needed. Two female specimens from Navassa (USNM 361059) are not Starksia sluiteri, as the markings in the second row of trunk blotches are elongate, not round.However, those markings are rectangular in the Navassa specimens, and the markings in the upper row are square—much more so than in our material of Starksia langi from the western Caribbean. The larger of the two females has some dark spots on the head as in Starksia langi. More material is needed. Other museum material examined (e.g., the UF specimens from Antigua and Mexico) are too faded to identify to species.

### Key to Species of the Starksia sluiteri Complex

**Table:** 

1a	Body with two rows of sharply contrasting dark blotches along sides of trunk, at least some markings in lower row vertically elongate; males without dark blotch in anterior portion of spinous dorsal fin, females with conspicuous round dark spots on head	Starksia langi (Belize, Honduras, Panama)
1b	Body with two or three rows of diffuse to sharply contrasting dark blotches along sides of trunk, those in second row mostly round; males with dark blotch in anterior portion of spinous dorsal fin, females with tiny dots but without conspicuous round dark spots on head	2
2a	Portions of head (at least cheek, operculum, gular region) with conspicuous pale round spots, this spotting pattern often much more prominent in males than females	Starksia greenfieldi (Tobago)
2b	Head without conspicuous pale round spots	Starksia sluiteri (Netherland Antilles)

## Discussion and conclusions

Gilbert (1965) and Greenfield (1979) noted that some species of Starksia can only be distinguished on the basis of color patterns—i.e., they exhibit no other morphological differences except sometimes modal differences in counts. Greenfield (1979) surmised that color patterns on the lips and sides of the head may be important in species recognition in blennioid fishes, which often live in cryptic habitats, in some cases (e.g., some chaenopsids) with only the heads typically visible. Our morphological investigation of the multiple genetic lineages within Starksia atlantica, Starksia lepicoelia, and Starksia sluiteri resulted in similar findings—i.e., most of the member species within the three complexes are distinguished from one another solely on the basis of pigment patterns, sometimes only differences in pigment on the lips and cheeks. All differences in counts are modal.

Morphological differences other than pigmentation separate some of the species complexes; for example, members of the Starksia atlantica complex lack an orbital cirrus, and those of Starksia lepicoelia have a scaled belly. Genetic divergence among species within each complex is generally smaller than that between complexes: 2–14% within Starksia atlantica, 1–9% within Starksia lepicoelia, and 7–19% within Starksia sluiteri vs. 17–22% between Starksia atlantica and Starksia lepicoelia, 17–24% between Starksia lepicoelia and Starksia sluiteri, and 17–23% between Starksia atlantica and Starksia sluiteri (Tables 4–7). The genetic distances separating species of the Starksia lepicoelia complex are particularly small, and those species are separated on the basis of minor differences in pigmentation on the head. Larger genetic distances separate most species of the Starksia sluiteri complex, and more prominent differences in trunk pigmentation separate some of those species. There is thus a correlation between small differences in COl sequences and minor differences in pigmentation, suggesting that pigment patterns may be among the first morphological changes accompanying speciation in Starksia. Greenfield (1979) did not have the benefit of genetic data for comparative purposes, but our COl data for four species in his Starksia ocellata complex (Fig. 1, Appendix 2) support his decision to recognize species almost entirely on the basis of minor differences in pigment. Although species recognition based on such limited morphological data may in general be a questionable practice, the congruence between Greenfield’s (1979) Starksia ocellata species and the COl data supports this practice in Starksia.

**Table 4.** Average (and range) Kimura two-parameter distance summary for the Starksia atlantica species complex based on cytochrome *c* oxidase l (COl) sequences of individuals represented in the neighbor-joining tree in Figure 1. Intraspecific averages are shown in bold. n/a = no average (one specimen). BAR – Barbados, SAB – Saba Bank, PAN – Panama.

Starksia	Starksia atlantica (n=7)	Starksia sangreyae A (n=6)	Starksia sangreyae B (n=6)	BAR(n=2)	SAB(n=1)	Starksia springeri (n=2)	PAN(n=2)
Starksia atlantica	1%(0-2)	-	-	-	-	-	-
Starksia sangreyae A	2%(2-3)	1%(0-2)	-	-	-	-	-
Starksia sangreyae B	2%(2-3)	2%(2-3)	1%(1-0)	-	-	-	-
BAR	9%(9-10)	10%(10-12)	9%(9-10)	0%(0)	-	-	-
SAB	9%(8-10)	9%(9-10)	9%(8-9)	3%(3)	n/an/a	-	-
Starksia springeri	9%(8-10)	10%(9-10)	9%(8-10)	5%(5-6)	5%(5)	0%(0)	-
PAN	13%(12-14)	13%(12-14)	13%(12-13)	11%(11)	11%(10-11)	12%(11-12)	0%(0)

**Table 5.** Average (and range) Kimura two-parameter distance summary for the Starksia lepicoelia species complex based on cytochrome *c* oxidase l (COl) sequences of individuals represented in the neighbor-joining tree in Figure 1. Intraspecific averages are shown in bold; n/a = no average (one specimen).

Starksia	Starksia lepicoelia A(n=7)	Starksia lepicoelia B(n=2)	Starksia robertsoni (n=3)	Starksia weigti (n=12)	Starksia williamsi (n=1)
Starksia lepicoelia A	1%(0-2)	-	-	-	-
Starksia lepicoelia B	5%(4-6)	1%(1)	-	-	-
Starksia robertsoni	7%(6-7)	7%(7-8)	0%(0-1)	-	-
Starksia weigti	6%(5-8)	6%(6-7)	2%(1-2)	0%(0-1)	-
Starksia williamsi	8%(8-9)	8%(8)	7%(7)	7%(7)	n/an/a

**Table 6.** Average (and range) Kimura two-parameter distance summary for the Starksia sluiteri species complex based on cytochrome *c* oxidase l (COl) sequences of individuals represented in the neighbor-joining tree in Figure 1. Intraspecific averages are shown in bold.

Starksia	Starksia greenfieldi (n=6)	Starksia fasciata (n=4)	Starksia sluiteri (n=4)	Starksia langi (n=6)
Starksia greenfieldi	1%(0-2)	-	-	-
Starksia fasciata	8%(7-9)	1%(0-2)	-	-
Starksia sluiteri	14%(13-14)	15%(14-17)	1%(0-1)	-
Starksia langi	17%(17-19)	16%(16-19)	16%(16-17)	1%(0-2)

**Table 7.** Range Kimura two-parameter distance summary for the Starksia atlantica, Starksia lepicoelia, and Starksia sluiteri species complexes based on cytochrome *c* oxidase l (COl) sequences of individuals represented in the neighbor-joining tree in Figure 1. Within-complex ranges are shown in bold.

	Starksia atlantica complex(n=26)	Starksia lepicoelia complex(n=25)	Starksia sluiteri complex(n=20)
Starksia atlantica complex	2-14%	-	-
Starksia lepicoelia complex	17-22%	1-9%	-
Starksia sluiteri complex	17-23%	17-24%	7-19%

There is not, however, universal congruence between genetic divergence and recognizable morphological differences in our data set. One Starksia greenfieldi specimen, TOB 9312, is 2% different from other Starksia greenfieldi, and one Starksia fasciata, TCI 9204, is 2% different from other Starksia fasciata. Both of those values are high for intraspecific variation in fishes in general (often well less than 1%), but we find no morphological evidence supporting the genetic divergences. Similarly Starksia sangreyae comprises two sublineages that are as genetically distinct in COl (2–3%) as Starksia sangreyae is from Starksia atlantica (2–3%), yet no consistent morphological differences were discovered, not even minor differences in color pattern. Even more puzzling, the two genetic sublineages of Starksia lepicoelia are4–6% different in COl, yet we found no morphological differences between them (Fig. 6). Very little material of one of those lineages is available, and further investigation is needed. Specimens in the two lineages were taken in the Bahamas at the same station, in 20–40 ft. of water off Great Stirrup Cay.

In contrast to the examples above, very little sequence divergence in COl exists between Starksia sangreyae from Belizeand Starksia atlantica from Bahamas/Turks and Caicos(2–3%), yet those species are easily distinguished on the basis of trunk pigment. Similar incongruences between COl data and morphology have been documented. For example, Baldwin et al. (2009b) found two morphological (pigment) variants of the goby Coryphopterus venezuelae, yet those morphs are not genetically distinct. Victor (2010) pointed out incongruences between COl data and morphologically recognizable species in greenbanded gobies (Elacatinus spp.). Specifically, he noted that Elacatinus multifasciatus from the eastern Caribbean and Elacatinus panamensis from Panama are morphologically extremely similar, but exhibit 11.3% sequence divergence in COl; he further noted that despite prominent differences in color pattern between Elacatinus rubrigenus and Elacatinus panamensis, those species exhibit only 3.3% sequence divergence in COl.

Those examples notwithstanding, the general congruence between COl lineages and morphologically recognizable species in western Atlantic Starksia is remarkable, and we have found the same to be true in our genetic and morphological investigations of other shorefish genera (e.g., Baldwin et al. 2009a, Baldwin et al. 2009b, Tornabene et al. 2010). A paper summarizing Smithsonian investigations of western Central Atlantic shorefish diversity and the utility of DNA Barcoding in this work is in preparation. Cases in which incongruences exist between genetic and morphological data ultimately will be further investigated; because DNA barcoding involves sequencing a relatively short segment of a single mitochondrial gene, adding additional genetic data may help resolve some conflicts. On the morphological side, adding information from early life history stages may be of value: the pelagic larval stages of many marine fishes offer a suite of characters for study not present in adults.

**Figure 16.**
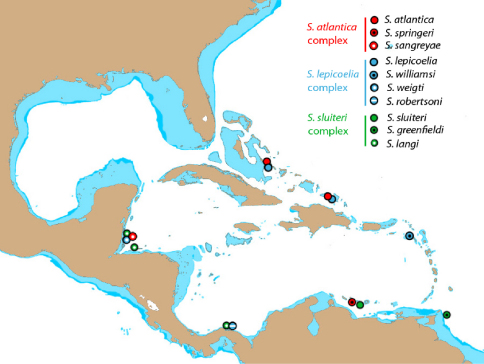
Distribution of species in the Starksia atlantica, Starksia lepicoelia, and Starksia sluiteri complexes. Only locations for genetically analyzed specimens plotted. Additional locations for some species discussed in text.

A striking element of our COl data for Starksia (Fig. 1) is the correlation between genetic lineages and geography within the Starksia atlantica, Starksia lepicoelia, Starksia sluiteri, and Starksia ocellata species complexes Specimens from Bahamas, Belize, Curacao, Saba Bank, and Tobago never occur in more than one genetic lineage within each complex, yet the species complexes themselves are broadly distributed (Fig. 16). Starksia nanodes also appears to be a broadly distributed species complex, with geographically distinct genetic lineages in Panama, Barbados, Saba Bank, and Belize (Fig. 1). Greenfield (1979) proposed superspecies status (sensu Amadon 1966, Mayr 1963) for the Starksia ocellata complex based on its six allopatric component species, and the Starksia atlantica, Starksia lepicoelia, and Starksia sluiteri species complexes described herein could be categorized likewise (we note, however, that the superspecies category has not been widely adopted in systematic treatments of fishes). It is not clear what evolutionary mechanisms are driving speciation within Starksia, but the life history of the group is characterized by a short pelagic phase of about two weeks (Victor, unpublished data). Although pelagic larval duration (PLD) is not always a good indicator of genetic structure (e.g., Bowen et al. 2006), a short PLD combined with restricted movement of adults may support the evolution of numerous allopatric species within a group by restricting gene flow among populations. It is premature to conduct a phylogeographic analysis of western Atlantic Starksia, but we concur with Greenfield (1979) that the division of some Starksia species into multiple allopatric component species is not typical of western Atlantic shorefishes in general. As noted by Floeter et al. (2008), Briggs’ (1974) two major biogeographic provinces of the Caribbean (western Caribbean plus Florida and West Indian/eastern Caribbean) are largely supported by recent genetic and biogeographical studies. Starksia is not the only exception to this general trend. Colin (2010) described five eco-morphological suites of western Atlantic Elacatinus goby species that are similar to our Starksia species complexes in that each comprises multiple species usually with allopatric distributions, and the suites themselves are broadly distributed. Considerably more studies of diversity and distribution of speciose genera of small, cryptic, Caribbean reef fishes and other Caribbean marine life are needed to determine if there are subdivisions of the major biogeographic provinces and, if so, what evolutionary mechanisms may be supporting them. Rocha et al. (2005) suggested that ecological speciation, in which natural selection in different environmental conditions in adjacent locations may drive populations along separate evolutionary pathways, could help explain high levels of species diversity in marine fishes in the absence of sufficient physical barriers to account for that diversity. Colin (2010) suggested that faunal breaks in Elacatinus species may correlate well with observed ocean currents, and he proposed to further investigate known fish distributions and actual dispersal potential as estimated from satellite-tracked current drifters.

For Starksia, future investigation must include more taxonomic and geographic coverage. Increased sampling will assuredly result in the recognition of new species and likely of new species complexes. The faunal breaks that separate members of the species complexes are unknown. In Starksia atlantica and Starksia lepicoelia, our specimens from Bahamas and Turks and Caicos represent the same species, and in Starksia sluiteri, specimens from Belize, Honduras, and Panama appear to be the same. Specimens in close proximity geographically thus tend to cluster into recognizable species. As better coverage is attained, it will be interesting to see if the same geographical boundaries characterize more than one of the species complexes or if the boundaries are different for each. Likewise it will be interesting to compare geographic boundaries of Starksia species with faunal breaks in other reef fishes such as Elacatinus. Future phylogenetic studies in which relationships among species and species complexes of Starksia and other groups are hypothesized should help shed light on patterns of speciation in small reef fishes of the western Atlantic.

Because we do not know how much more investigation is required to obtain a reasonably complete picture of Starksia biodiversity and biogeography, the words of Winston Churchill included as an epigraph in this paper seem particularly appropriate. The study of Starksia must continue.

## Supplementary Material

XML Treatment for Starksia sangreyae

XML Treatment for Starksia springeri

XML Treatment for Starksia

XML Treatment for Starksia weigti

XML Treatment for Starksia williamsi

XML Treatment for Starksia robertsoni

XML Treatment for Starksia greenfieldi

XML Treatment for Sarksia langi

## Appendix 1

Starksia specimens included in the genetic analysis (Fig. 1) but not examined for the species accounts. The voucher specimens from Barbados (BAR), Brazil (BRZ), Panama (PAN), Florida (FLA 090), and St. Thomas (STVI) are part of B. Victor’s personal collection.

**Table:**

Species	DNA	Standard Length (mm)	Specimen voucher
Starksia atlantica	PAN 091	9.2	PAN-LIDB091
		PAN 092	9.3	PAN-LIDB092
		BAR 009	9.9	BAR-LIDB009
		BAR 062	9.1	BAR-LIDB062
Starksia occidentalis	BLZ 6271	17.5	USNM 399661
		BLZ 6273	30	USNM 399662
		BLZ 6304	27	No
		BLZ 7335	13	USNM 399663
		BLZ 7749	25	USNM 399664
		BLZ 8248	24	USNM 399669
		BLZ 8249	27	USNM 399670
		BLZ 8250	23	USNM 399671
		BLZ 8317	25	USNM 399672
		BLZ 8375	33	USNM 399665
		BLZ 8376	30	USNM 399666
		BLZ 8377	9	No
		BLZ 8378	15	USNM 399667
		BLZ 8379	22	USNM 399668
		PAN 058	19.2	PAN-BVCOR058
Starksia ocellata	FLA 090	8.3	FLA-LIDB090
		FLA 7391	35	USNM 399686
Starksia guttata	TOB 9016	25	USNM 399630
		TOB 9017	25	USNM 399631
		TOB 9018	23.5	USNM 399632
		TOB 9127	11.5	USNM 399633
Starksia culebrae	STVI 1336	20	USVI-LIDM1336
		STVI 019	16	USVI-LIDB019
		STVI 121	30.4	USVI-LIDMA121
Starksia elongata	TCI 9115	24	USNM 399634
Starksia starcki	BLZ 8297	11	USNM 399660
Starksia hassi	CUR 8096	28	USNM 399627
		CUR 8097	24	USNM 399628
		CUR 8319	19	USNM 399629
		CUR 8320	10	No
		CUR 8349	10	No
Starksia multilepis	BRZ 1323	23.2	NOR-LIDM1323
		BRZ 1324	19.3	NOR-LIDM1324
		BRZ 1325	19.4	NOR-LIDM1325
Starksia nanodes	BAR 011	9	BAR-LIDB011
		BAR 013	12.9	BAR-LIDB013
		BLZ 5076	12	No
		BLZ 5104	13	USNM 399673
		BLZ 5105	14	USNM 399674
		BLZ 5162	15	USNM 399675
		BLZ 5163	12	USNM 399676
		BLZ 5277	8	No
		BLZ 5424	15.5	USNM 399677
		BLZ 6124	13	USNM 399678
		BLZ 6125	13	USNM 399679
		BLZ 7330	8.5	No
		BLZ 7680	15	No
		BLZ 8121	15	USNM 399680
		BLZ 8289	8.5	No
		BLZ 8391	12.5	USNM 399954
		PAN 053	14.8	PAN-BVCOR053
		PAN 054	13.3	PAN-BVCOR054
		PAN 056	14	PAN-BVCOR056
		PAN 1247	18.4	PAN-LIDM1247
		SAB 0606056	NA *	USNM 397404

## Appendix 2

Average (and range) Kimura two-parameter distance summary for Starksia based on cytochrome *c* oxidase l (COl) sequences of all individuals represented in the neighbor-joining tree in Figure 1. Intraspecific averages are shown in bold. n/a = no average (one specimen). BAR – Barbados, SAB – Saba Bank, PAN – Panama, BLZ – Belize.

**Table:**

Starksia species		1	2	3	4	5	6	7	8	9	10	11	12	13	14	15	16	17	18	19	20	21	22	23	24	25	26	27	28	29
Starksia greenfieldi (n = 6)	1	1% (0-2)	-	-	-	-	-	-	-	-	-	-	-	-	-	-	-	-	-	-	-	-	-	-	-	-	-	-	-	-
Starksia fasciata (n = 4)	2	8% (7-9)	1% (0-2)	-	-	-	-	-	-	-	-	-	-	-	-	-	-	-	-	-	-	-	-	-	-	-	-	-	-	-
Starksia sluiteri (n = 4)	3	14% (13-14)	15% (14-17)	1% (0-1)	-	-	-	-	-	-	-	-	-	-	-	-	-	-	-	-	-	-	-	-	-	-	-	-	-	-
Starksia langi (n = 6)	4	17% (17-19)	16% (16-19)	16% (16-17)	1% (0-2)	-	-	-	-	-	-	-	-	-	-	-	-	-	-	-	-	-	-	-	-	-	-	-	-	-
Starksia multilepis (n = 3)	5	23% (22-24)	21% (20-21)	21% (21)	17% (17-18)	0% (0)	-	-	-	-	-	-	-	-	-	-	-	-	-	-	-	-	-	-	-	-	-	-	-	-
Starksia hassi (n = 5)	6	23% (22-25)	21% (21-22)	22% (22-23)	21% (21-22)	22% (21-22)	1% (0-1)	-	-	-	-	-	-	-	-	-	-	-	-	-	-	-	-	-	-	-	-	-	-	-
Starksia occidentalis (n = 15)	7	23% (22-24)	23% (22-24)	22% (21-23)	21% (21-22)	20% (20-21)	21% (20-22)	0% (0-1)	-	-	-	-	-	-	-	-	-	-	-	-	-	-	-	-	-	-	-	-	-	-
Starksia ocellata (n = 2)	8	22% (22-23)	22% (22-23)	21% (20-22)	21% (21-22)	20% (20)	22% (21-22)	3% (3-3)	0% (0)	-	-	-	-	-	-	-	-	-	-	-	-	-	-	-	-	-	-	-	-	-
Starksia guttata (n = 4)	9	21% (20-22)	21% (21-22)	19% (18-20)	21% (20-22)	21% (20-21)	19% (18-20)	4% (3-4)	5% (5)	1% (0-1)	-	-	-	-	-	-	-	-	-	-	-	-	-	-	-	-	-	-	-	-
Starksia culebrae (n = 4)	10	23% (23-24)	24% (23-24)	21% (20-21)	22% (21-24)	20% (20)	21% (21-22)	6% (5-6)	6% (5-6)	6% (5-6)	0% (0)	-	-	-	-	-	-	-	-	-	-	-	-	-	-	-	-	-	-	-
Starksia elongata (n = 1)	11	20% (19-20)	20% (19-20)	20% (19-21)	19% (18-20)	21% (21)	23% (22-24)	16% (15-16)	15% (15-16)	16% (16)	17% (17)	n/a n/a	-	-	-	-	-	-	-	-	-	-	-	-	-	-	-	-	-	-
Starksia lepicoelia A (n = 7)	12	20% (19-21)	20% (19-21)	19% (18-20)	22% (21-22)	22% (22-23)	23% (22-24)	19% (18-20)	18% (17-19)	19% (17-20)	19% (18-19)	24% (23-24)	1% (0-2)	-	-	-	-	-	-	-	-	-	-	-	-	-	-	-	-	-
Starksia lepicoelia B (n = 2)	13	20% (19-21)	20% (20-21)	18% (17-19)	21% (20-22)	23% (22-23)	22% (22-23)	18% (18)	19% (18-19)	18% (17-18)	18% (18)	23% (22-23)	5% (4-6)	1% (1)	-	-	-	-	-	-	-	-	-	-	-	-	-	-	-	-
Starksia robertsoni (n = 3)	14	21% (20-22)	22% (22-23)	20% (19-21)	21% (20-22)	22% (22)	23% (22-23)	19% (19-20)	19% (19-20)	20% (19-21)	19% (18-19)	22% (22)	7% (6-7)	7% (7-8)	0% (0-1)	-	-	-	-	-	-	-	-	-	-	-	-	-	-	-
Starksia weigti (n = 12)	15	20% (19-23)	22% (22-24)	20% (19-20)	21% (20-22)	22% (22-23)	22% (21-23)	19% (19-20)	19% (18-19)	20% (19-20)	19% (19-20)	23% (22-24)	6% (5-8)	6% (6-7)	2% (1-2)	0% (0-1)	-	-	-	-	-	-	-	-	-	-	-	-	-	-
Starksia williamsi (n = 1)	16	20% (20-21)	21% (21)	20% (20-20)	21% (21-22)	22% (22)	20% (19-20)	19% (19-20)	19% (19)	20% (20-21)	19% (19)	23% (23)	8% (8-9)	8% (8)	7% (7)	7% (7)	n/a n/a	-	-	-	-	-	-	-	-	-	-	-	-	-
BAR (n = 3)	17	20% (19-21)	20% (19-21)	22% (21-23)	21% (20-21)	23% (23)	22% (21-22)	21% (20-22)	21% (21-22)	20% (19-21)	21% (21-22)	21% (21-22)	14% (13-15)	15% (15)	14% (14-15)	13% (13-15)	13% (13-14)	1% (1)	-	-	-	-	-	-	-	-	-	-	-	-
Starksia starcki (n = 1)	18	23% (23-24)	24% (23-25)	24% (24)	22% (22-24)	26% (26)	25% (25-26)	22% (22-23)	22% (22)	23% (23-24)	24% (24)	23% (23)	24% (24-25)	25% (25)	24% (24)	24% (23-25)	24% (24)	25% (24-26)	n/a n/a	-	-	-	-	-	-	-	-	-	-	-
Starksia atlantica (n = 7)	19	19% (19-21)	21% (20-22)	22% (21-22)	20% (20-22)	21% (20-22)	22% (21-23)	21% (20-21)	20% (20-21)	20% (20-21)	20% (19-21)	21% (20-21)	19% (18-20)	18% (18-19)	18% (17-19)	18% (17-20)	19% (18-20)	22% (21-23)	22% (21-23)	1% (0-2)	-	-	-	-	-	-	-	-	-	-
Starksia sangreyae A (n = 6)	20	20% (19-21)	20% (19-21)	22% (21-23)	21% (20-22)	22% (21-22)	23% (22-24)	21% (21-22)	20% (20-21)	21% (20-22)	19% (19-20)	21% (21-22)	19% (19-20)	19% (18-20)	18% (17-18)	18% (17-21)	18% (18)	22% (21-22)	22% (22-23)	2% (2-3)	1% (0-2)	-	-	-	-	-	-	-	-	-
Starksia sangreyae B (n = 6)	21	20% (19-22)	21% (20-22)	22% (22-23)	20% (19-20)	21% (21-22)	23% (22-24)	21% (21-22)	21% (20-21)	21% (21-22)	20% (20-21)	21% (20-21)	20% (19-21)	20% (19-20)	18% (17-18)	18% (17-20)	19% (19-20)	22% (21-23)	22% (22-23)	2% (2-3)	2% (2-3)	1% (1-0)	-	-	-	-	-	-	-	-
BAR (n = 2)	22	19% (18-20)	20% (20)	20% (20-21)	18% (17-19)	23% (23)	20% (19-20)	20% (20)	20% (20)	19% (18-19)	19% (19)	20% (20)	21% (20-21)	19% (19-20)	18% (18)	19% (18-21)	19% (19)	22% (21-23)	22% (22)	9% (9-10)	10% (10-12)	9% (9-10)	0% (0)	-	-	-	-	-	-	-
SAB (n = 1)	23	19% (18-20)	20% (20-21)	21% (20-22)	18% (18-19)	24% (24)	20% (19-20)	20% (20-21)	20% (20)	19% (19)	19% (19)	19% (19)	20% (20-21)	19% (19)	19% (18-19)	19% (18-20)	18% (18)	22% (21-22)	21% (21)	9% (8-10)	9% (9-10)	9% (8-9)	3% (3)	n/a n/a	-	-	-	-	-	-
Starksia springeri (n = 2)	24	18% (17-19)	20% (20-21)	21% (20-21)	19% (18-20)	24% (24)	23% (20-21)	19% (19-20)	19% (19-20)	18% (18-19)	18% (18-19)	18% (18)	21% (20-21)	19% (19-20)	18% (18-19)	19% (19-21)	18% (18)	21% (21-22)	21% (20-21)	9% (8-10)	10% (9-10)	9% (8-10)	5% (5-6)	5% (5)	0% (0)	-	-	-	-	-
PAN (n = 2)	25	20% (20-21)	21% (20-21)	22% (21-23)	20% (20-21)	23% (23-24)	23% (22-24)	18% (18-19)	19% (19)	19% (18-19)	18% (18-19)	21% (21)	21% (20-22)	20% (20)	20% (19-20)	20% (19-20)	19% (19)	21% (21)	25% (25-26)	13% (12-14)	13% (12-14)	13% (12-13)	11% (11)	11% (10-11)	12% (11-12)	0% (0)	-	-	-	-
Starksia nanodes PAN (n = 5)	26	24% (23-25)	22% (21-23)	22% (21-23)	21% (20-22)	21% (20-22)	20% (20-21)	23% (22-)	23% (22-23)	22% (21-23)	23% (22-24)	23% (23)	22% (21-23)	22% (21-22)	22% (22-23)	23% (22-24)	22% (22)	24% (23-24)	27% (26-27)	19% (18-20)	20% (19-21)	20% (19-21)	20% (20-21)	20% (20-21)	23% (22-23)	22% (22-23)	1% 0(1-)	-	-	-
Starksia nanodes SAB (n = 1)	27	24% (24-25)	24% (23-24)	23% (22-23)	21% (21)	21% (21-21)	25% (25-26)	22% (22-24)	22% (22-22)	23% (23-24)	21% (21)	24% (24)	23% (22-24)	22% (22-23)	22% (22)	22% (22-23)	23% (23)	24% (24-25)	29% (29)	20% (19-20)	20% (19-20)	21% (20-21)	20% (19-20)	20% (20)	23% (22-23)	20% (20)	12% (11-12)	n/a n/a	-	-
Starksia nanodes BAR (n = 1)	28	24% (24-25)	24% (24-25)	22% (22-23)	20% (20-21)	22% (22)	25% (25-26)	23% (23-23)	23% (22-23)	24% (23-24)	22% (22)	25% (25)	24% (23-24)	21% (21)	23% (22-23)	24% (23-24)	23% (23)	25% (24-25)	29% (29)	19% (19-20)	20% (19-21)	20% (20-20)	20% (20)	19% (19)	22% (21-22)	21% (21)	11% (10-11)	3% (3)	n/a n/a	-
Starksia nanodes BLZ (n = 14)	29	25% (24-28)	23% (22-24)	22% (21-24)	21% (20-22)	20% (20-21)	25% (24-26)	23% (22-25)	23% (22-24)	23% (22-24)	24% (23-25)	22% (21-22)	23% (22-25)	22% (21-23)	24% (23-25)	23% (22-25)	23% (24-25)	22% (22-23)	26% (26-28)	18% (17-19)	19% (18-19)	19% (18-19)	21% (20-22)	21% (20-22)	20% (20-21)	21% (21-23)	12% (11-14)	11% (11-12)	11% (11-12)	0% (0-1)
